# Genomic Adaptations to an Endoparasitic Lifestyle in the Morphologically Atypical Crustacean *Sacculina carcini* (Cirripedia: Rhizocephala)

**DOI:** 10.1093/gbe/evac149

**Published:** 2022-10-12

**Authors:** Sebastian Martin, Peter Lesny, Henrik Glenner, Jochen Hecht, Andreas Vilcinskas, Thomas Bartolomaeus, Lars Podsiadlowski

**Affiliations:** Centre for Molecular Biodiversity Research (zmb), Zoological Research Museum Alexander Koenig (ZFMK), LIB, Bonn, Germany; Department of Comparative Ultrastructure and Evolution of Invertebrates, Institute for Evolutionary Biology & Animal Ecology, University Bonn, Bonn, Germany; Department of Comparative Ultrastructure and Evolution of Invertebrates, Institute for Evolutionary Biology & Animal Ecology, University Bonn, Bonn, Germany; Department of Biol. Sciences, University Bergen, Bergen, Norway; Centre for Macroecology, Evolution and Climate, Copenhagen, Denmark; Genomics Unit, Centre for Genomic Regulation (CRG), The Barcelona Institute of Science and Technology, Barcelona, Spain; Department of Insect Biotechnology, Justus-Liebig-University of Giessen, Giessen, Germany; Department of Bioressources, Fraunhofer Institute for Molecular Biology and Applied Ecology, Giessen, Germany; Department of Comparative Ultrastructure and Evolution of Invertebrates, Institute for Evolutionary Biology & Animal Ecology, University Bonn, Bonn, Germany; Centre for Molecular Biodiversity Research (zmb), Zoological Research Museum Alexander Koenig (ZFMK), LIB, Bonn, Germany; Department of Comparative Ultrastructure and Evolution of Invertebrates, Institute for Evolutionary Biology & Animal Ecology, University Bonn, Bonn, Germany

**Keywords:** endoparasite, genome, *sacculina*, rhizocephala, differential expression

## Abstract

The endoparasitic crustacean *Sacculina carcini* (Cirripedia: Rhizocephala) has a much simpler morphology than conventional filter-feeding barnacles, reflecting its parasitic lifestyle. To investigate the molecular basis of its refined developmental program, we produced a draft genome sequence for comparison with the genomes of nonparasitic barnacles and characterized the transcriptomes of internal and external tissues. The comparison of clusters of orthologous genes revealed the depletion of multiple gene families but also several unanticipated expansions compared to non-parasitic crustaceans. Transcriptomic analyses comparing interna and externa tissues revealed an unexpected variation of gene expression between rootlets sampled around host midgut and thoracic ganglia. Genes associated with lipid uptake were strongly expressed by the internal tissues. We identified candidate genes probably involved in host manipulation (suppression of ecdysis and gonad development) including those encoding crustacean neurohormones and the juvenile hormone binding protein. The evolution of Rhizocephala therefore appears to have involved a rapid turnover of genes (losses and expansions) as well as the fine tuning of gene expression.

**Significance**

The genome of the endoparasitic crustacean *Sacculina carcini* was sequenced. Although morphologically extremely simplified, the genome of *S. carcini* is of similar complexity than genomes of other crustaceans. We could identify a few candidate genes for host manipulation.

## Introduction

Among the diverse morphological and life history strategies of the arthropods, barnacles (Cirripedia) exemplify some of the most striking deviations from the arthropod ground pattern (Anderson 1993). All barnacles are sessile as adult organisms, permanently attached to substrates that are often taxon-specific. Most barnacles rely on suspension feeding, in which six pairs of highly modified thoracic appendages are used to filter food particles from seawater. However, adult members of Rhizocephala are parasites with little morphological resemblance to their suspension-feeding relatives or other arthropods. The dispersal stages of all major clades of barnacles (Acrothoracica, Thoracica, and Rhizocephala) are nauplius larvae (as typical of many crustaceans), but the last pelagic nauplius stage is followed by a final settling stage unique to the barnacles, known as the cypris larva. This larval type can swim fast and directionally but is also capable of bipedal walking on a pair of modified antennules that determine whether the substrate is suitable for settling. These mobile larval stages were the main characters that convinced Thompson (Thompson 1830) that barnacles should be considered as arthropods and not, as was hitherto thought, mollusks. Charles Darwin spent several years studying barnacles, culminating in four monographs on extant and fossil species (Deutsch 2009).

**Fig. 1.**
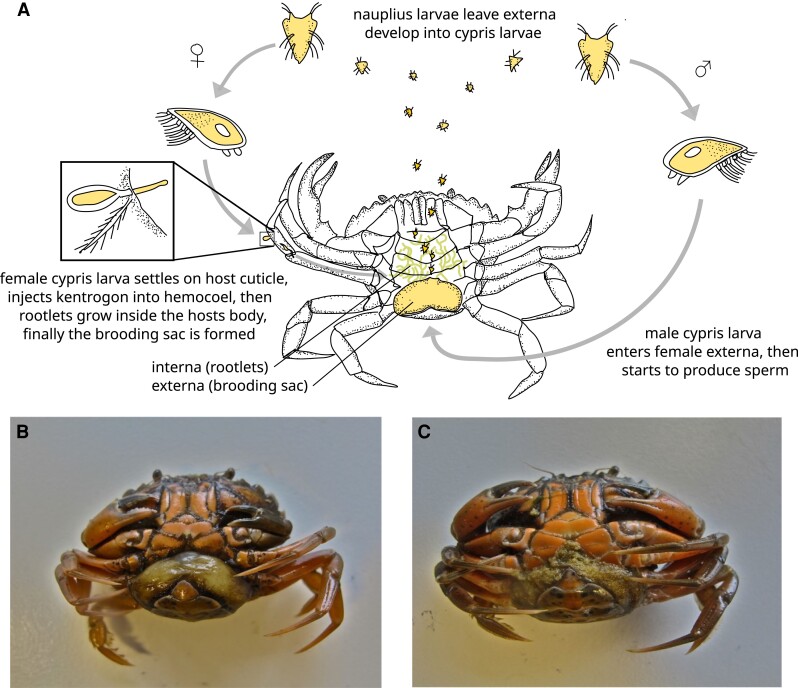
Life cycle of *Sacculina carcini* (*A*). Host crab Carcinus maenas with externa from *S. carcini* under the pleon, “mimicking” egg mass (*B*). Gravid female crab with eggs (*C*).

Although the characteristics of Rhizocephala larvae confirm they belong to the barnacles, their endoparasitic lifestyle has led to extreme morphological reduction (Walker 2001). Their cypris larval stage settles on the host (usually a decapod crustacean), but rather than undergoing metamorphosis on the surface it injects an infectious stage into the host's body (fig. 1*A*). Rhizocephala adults possess a radically simplified morphology, lacking appendages, complex sensory organs, mouth, gut, respiratory, and excretory organs, providing an extreme example of reduced morphological complexity unique among arthropods (Anderson 1993; Høeg and Lutzen 1995; Høeg 1995; Glenner 2001; Walker 2001). The parasitic barnacles were named Rhizocephala by Müller (Müller 1862). But in the original description, only the external brooding sac was identified, whereas the internal rootlet tissue infiltrating the host's body was later described thoroughly by Anderson (Anderson 1884). By now, approximately 250 species have been reported, and their parasitic lifestyle probably dates to a last common ancestor living about 200 million years ago, making them one of the oldest endoparasitic lineages among multicellular animals (Pérez-Losada et al. 2008).

*Sacculina carcini* is a member of the Rhizocephala that parasitizes the green shore crab *Carcinus maenas*, which is common on the European marine coast from Spain to northern Scandinavia. Like all rhizocephalans, adult specimens of *S. carcini* feature two distinct and morphologically simple body parts—the externa and the interna (Høeg 1995). The externa is the outer part of a female parasite, visible on the carapace of the host as a yellow to brownish sac-like structure. It is located under the pleon of the host where non-parasitized female crabs carry their eggs (fig. 1*B*, 1*C*) and comprises the reproductive apparatus of the parasite including ovary, collecteric glands and a mantle cavity which store the developing nauplius larvae until they are released to the ambient sea water. Males are present in the form of one to two dwarf males placed and maintained by the female in a pair of so-called receptacles close to the ovary. Here they serve as functional testis, producing the sperm that fertilize the eggs (Høeg and Lutzen 1995). The exploitative lifestyle of such “dwarf males” was proposed as a prerequisite for the evolution of females into endoparasites that exploit other hosts (Scholtz et al. 2009). The externa connects, via a stalk that penetrates the cuticle of the host, to a root-like structure, the interna (Delage 1884), that is embedded in host hemocoel and host tissue and acts as the trophic organ of the parasite. The extensive rootlet system of the interna infiltrates several organs of the host and consists of a single layer of epithelial cells covered by an extremely thin epicuticle, which probably takes up nutrients from the hosts hemolymph.

An infestation with *S. carcini* has striking effects on host behavior and morphology (Mouritsen and Jensen 2006; Kristensen et al. 2012): Host crabs are castrated and individuals of both sexes behave like a gravid female crab—grooming the externa as if it was the crab's own egg mass. Infested male crabs show, in addition, partial morphological feminization and in both sexes, growth is prevented by the arrest of the moulting cycle. Parasitized crabs are mainly found submerged in seawater, whereas nonparasitized crabs sometimes forage on shallow water and tideland, suggesting additional behavioral changes (Rasmussen 1959). Several detailed studies have focused on the morphological aspects of Rhizocephala, including *S. carcini* (Bresciani and Høeg 2001; Glenner 2001; Miroliubov et al. 2020). These have shown that the rootlet system can damage host tissue during infiltration, leading to necrosis and partial organ degeneration (Powell and Rowley 2008; Rowley et al. 2020). However, genetic data on the Rhizocephala are sparse, restricted to phylogenetic and population genetic studies with standard markers (Pérez-Losada et al. 2004; Glenner and Hebsgaard 2006; Gurney et al. 2006), and a small number of publications concerning the expression of *hox* and *engrailed* genes during *S. carcini* larval development (Queinnec et al. 1999; Mouchel-Vielh et al. 1998, 2002; Gibert et al. 2000; Rabet et al. 2001; Deutsch and Mouchel-Vielh 2003; Géant et al. 2006). A single phylogenomic study has also been published, based on the sequences of nuclear genes from *Loxothylacus* (Regier et al. 2010).

The first barnacle genome sequences were recently made available, representing the non-parasitic acorn barnacle species *Amphibalanus amphitrite* (Kim et al. 2019) and *Semibalanus balanoides* (Nunez et al. 2021), as well as the goose neck barnacle *Pollicipes pollicipes* (Bernot et al. 2022). To address the lack of molecular data underpinning the simplified morphology and endoparasitic lifestyle of Rhizocephala, we sequenced the genome of *S. carcini* to compare its complexity with conventional barnacles and to document the amount of gene turnover during evolution. We also carried out transcriptome sequencing to characterize gene expression in the rootlets, an evolutionary novelty, to gain insight into how *S. carcini* might exploit and manipulate its host effectively.

## Results and Discussion

### Genome Assembly and Protein-coding Genes

The *S. carcini* draft genome was assembled from a combination of Oxford Nanopore (ONT) long reads and Illumina short reads. The total assembly size was 298.35 Mb, consisting of 13,055 scaffolds (scaffold N50 = 161 kb; contig N50 = 124 kb), 58% of which was made up of repetitive DNA. Assembly size may underestimate genome size due to 1) collapsed regions with tandem repeats and 2) the difficulties to assembly heterochromatic and simple repeat regions. Long read coverage of contigs and scaffolds outside of repeat regions is around 35x, suggesting that the assembly represents a haploid assembly.

**Fig. 2.**
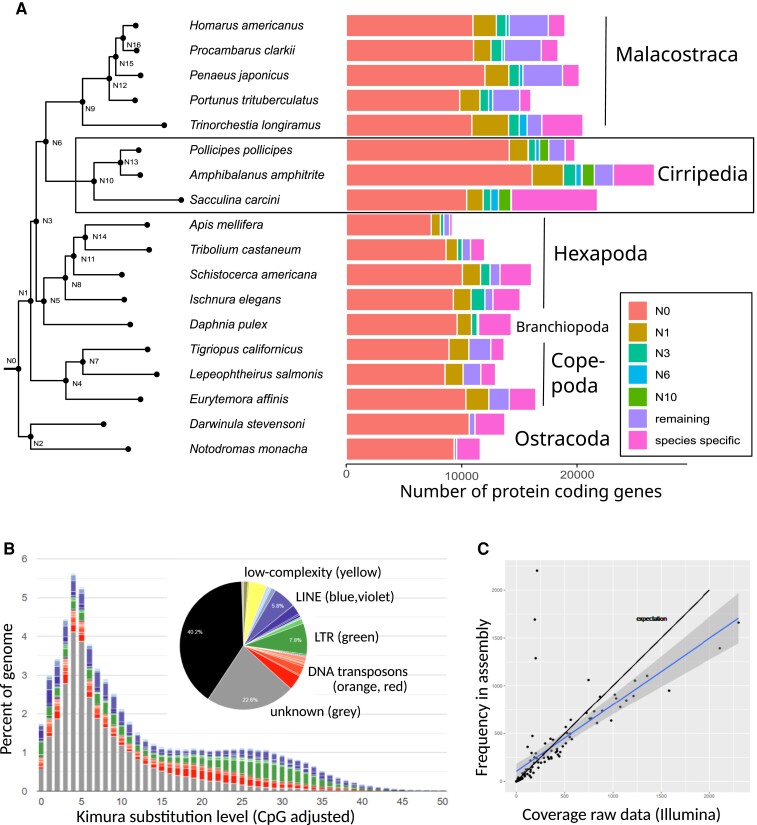
(*A*) Phylogenetic tree of arthropods obtained from concatenated single-copy ortholog alignments and phylostratigraphic occurrence of orthologs shared with other species or unique to that species. (*B*) Repeat landscape and percentage of repeat groups in the genome of *S. carcini.* (*C*) Correlation of the coverage of the 100 most frequent repeat families in the assembly and in short read raw data.

Gene annotation based on evidence from transcriptomic data and proteins from other crustaceans indicated a total of 25,117 protein-coding genes, which is close to the number predicted in the other barnacle genomes, namely *A. amphitrite* (25,580) and *P. pollicipes* (27,080) and well within the range predicted for other crustacean genomes (15,000–30,000) (fig. 2*A*). BUSCO analysis to identify universal single-copy orthologs (Seppey et al. 2019) of arthropods revealed the presence of orthologs for most conserved single-copy arthropod genes. The percentage of missing sequences (8.3%) is higher than in other cirripeds, namely *A. amphitrite* (1.8%) and *P. pollicipes* (4.5%). We found that 12.8% of the protein-coding sequences expected to be single-copy orthologs were duplicated in our predicted set of proteins. For 4.9% of orthologs we only found fragments among the predicted proteins. In comparison, *Drosophila melanogaster* and *Tribolium castaneum* preserve almost all conserved single-copy genes for arthropods, with a small percentage of duplicates and fragments. This is likely to reflect the more refined status of these well-studied and annotated model organism genomes and the fact that insect genomes are more prominently featured than crustacean genomes in the BUSCO reference sets.

The phylogenetic tree reconstructed from the orthofinder-predicted 1011 one-to-one ortholog alignments (fig. 2*A*) resembles a recently published topology for (pan)crustacean interrelationships (Schwentner et al. 2018), except for the position of Copepoda. In our tree, which was rooted with Ostracoda, we found that copepods were placed as sister group to a large clade comprising Malacostraca, Cirripedia, Branchiopoda, and Hexapoda. In the phylogenetic tree of Schwentner et al. (2018) Thecostraca (including Cirripedia) and Malacostraca were sister groups, which together form the sister group to the Copepoda. In our tree Branchiopoda and Hexapoda are sister groups, but we did not include any data from the Remipedia or Cephalocarida due to the lack of genomic information for these taxa. The tree was constructed to analyze the representation of orthologous protein-coding gene clusters and was not intended to evaluate the broader phylogenetic relationships between different crustacean taxa.

### Repeat Content

After analyzing repeat content and regions of low complexity, 58.8% of the assembly was masked, the majority due to similarity to complex repeats: repeat elements derived from DNA transposons (9.9%), retrotransposons (22.6%), and a high proportion of unclassified elements (22.6%) representing ∼55% of the genome assembly (fig. 2*B*). Approximately 5% was marked as low complexity and simple repeat regions. The repeat landscape shows that there is moderate recent activity in transposons, with the peak of activity in the past (repeat families with Kimura distance of 4 in between clusters).

An analysis comparing real genomic short reads and artificial reads derived from our assembly using deviaTE (Weilguny and Kofler 2019) revealed a discrepancy of variation and amounts for the top 100 most abundant repeat elements (fig. 2*C*). This suggests a larger extent of repeat regions in the real genome and probably collapsed tandem repeat regions in our assembly and missing repeats between the scaffolds, at least for some families.

### Orthologous Gene Clusters, Gene Duplications and Gene Loss

**Fig. 3.**
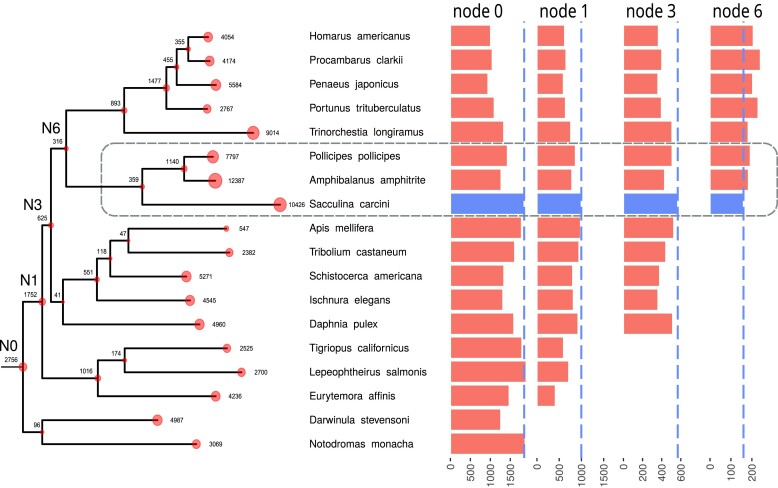
Gene duplications and gene loss. The numbers on the tree are duplication events among orthogroups in the specific species or node as presumed by orthofinder analysis. The barplot on the right side show the number of presumed gene loss of genes assumed to be present in the last common ancestor of the nodes 0, 1, 3, and 6 of the tree.

Phylostratigraphic occurrence of genes (fig. 2*A*), gene duplications, and gene loss (fig. 3) among the predicted protein sets of 18 crustacean and insect species were analyzed with orthofinder (Emms and Kelly 2019). We omitted all but one (=the longest) isoforms before protein sets were compared. Among the taxa analysed *S. carcini* has the highest number of “private” genes that cannot be associated with orthologs from other taxa. This is clearly a function of the distance to the next related species among those analysed, but even when compared to similarly isolated branches, e.g. *Trinorchestia longiramus*, the number is much higher (fig. 2*A*). The presumed number of gene duplications in orthogroups is high among all barnacles, especially in *A. amphitrite* and *S. carcini* (fig. 3), but also at the base of *A. amphitrite* and *P. pollicipes*.

Given the varying numbers of sequences present in the conserved clusters, we evaluated the number of orthologs expected but not present in a species, assuming these genes were present in the last common ancestor of a clade but subsequently lost somewhere in the lineage towards this species (fig. 3). Here, *S. carcini* has a high number of genes lost with respect to the common ancestor of the root of our tree (node 0, comprising crustaceans and insects) and of node 1 (all crustaceans and insects, except ostracods). Comparable high number of gene loss has also occurred in the copepods and *Apis mellifera*.

All these results must be taken with caution, because they are heavily affected by differences in annotation quality and by failures to detect homology in older protein families (Vakirlis et al. 2020; Weisman et al. 2020). We exclude the possibility that a large proportion of the genome is missing by 1) good BUSCO values with comparable percentage of missing genes with other the other barnacle genomes and 2) good success in mapping of Illumina reads from genomic DNA (97%) and from RNA (99%) to our long-read based assembly.

### Protein Family Expansions and Contractions

We investigated evolutionary changes in the size of protein families in *S. carcini* compared to *A. amphitrite*, *P. pollicipes*, and other crustaceans (*Penaeus vannamei, Hyalella azteca, L. salmonis*, and *Eurytemora affinis*) revealing significant differences in 305 cases. Contrary to our expectations, we found that 225 of these cases involved expansions in *S. carcini* (with 1043 genes, compared to an average of 326 in the other five species and 265 in *A. amphitrite*) while only 80 represented contractions (with 122 genes, compared to an average of 591 in the other five species, and 970 in *A. amphitrite*). The two most strikingly expanded gene families in *S. carcini* involve genes with protein kinase domains, which regulate many intracellular signaling pathways involved in environmental responses, metabolism and development (supplementary tables S3 and S4, Supplementary Material online).

### The *hox* Gene Cluster

The *hox* gene cluster plays a key role in the organization of the embryonic body plan in all metazoan species and is therefore a primary target for analysis when considering arthropods with atypical morphology (Hughes and Kaufman 2002). Changes in *hox* gene expression have been proposed to explain the derived body pattern of barnacles (Mouchel-Vielh et al. 1998; Deutsch and Mouchel-Vielh 2003) Interestingly, although the presence of a single *hox* cluster has been confirmed in *S. carcini*, the pivotal *abdominal-A* (*abd-A*) gene is either missing or not expressed in early embryos and has been invoked as the mechanisms underlying the “loss” of the barnacle abdomen (Géant et al. 2006).

**Fig. 4.**
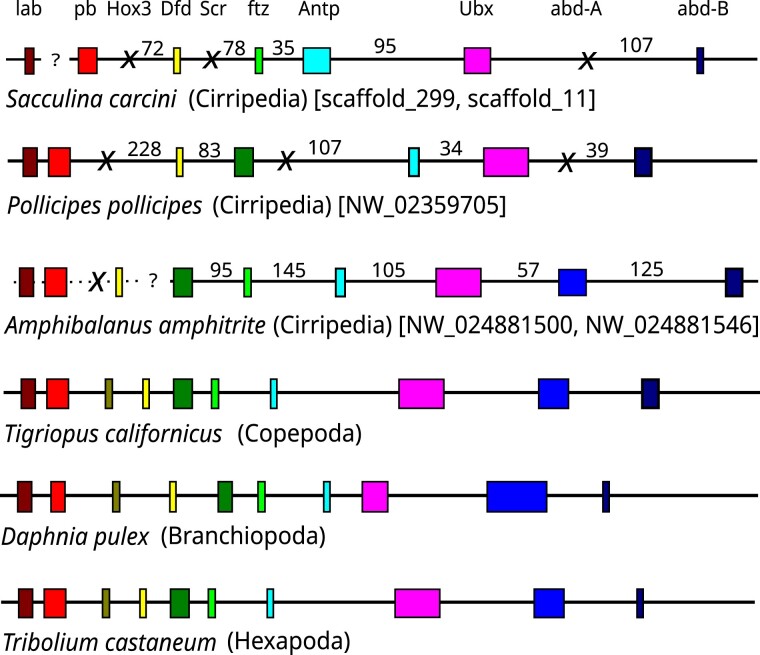
Analysis of the hox gene clusters in Cirripedia compared to other arthropods. Numbers depict size of intergenic regions (kbp).

We identified most of the *hox* genes by searching for the homeodomain and using a tree-based approach for comparison with other arthropods. The classical *hox* genes, expressed in early development, were found as a cluster on one of the larger scaffolds (fig. 4). The cluster contained all *hox* genes found in other crustaceans, except *labial* (*lab*), *Hox3/zerknüllt* (*Hox3*), *Sex combs reduced* (*Scr*) and Abdominal-A (*abd-A*). We found *lab* to be placed at the end of another scaffold, so it may be present on the same chromosome close to the rest of the cluster. In contrast, *Hox3*, *Scr*, and *abd-A* were missing from the regions where they are found in other arthropods, with *abd-A* usually located between Ultrabithorax (*Ubx*) and *abdominal-B* (*abd-B*). We carefully inspected the regions where these genes are missing in *S. carcini*, involving blast searches on nucleotide and aminoacid level but found no evidence for remnants of the lost genes here.

A homeobox containing gene which is phylogenetically placed in the *abd-A* cluster (supplementary fig. S1, Supplementary Material online; XP_043214087.1 homeobox protein Hox-C6-like) was identified in *A. amphitrite*, placed between *Ubx* and *abd-B* in the usual position of the arthropod hox cluster. However, the abdominal-a domain (PFAM12407), which usually accompanies the homeodomain is truncated here to half of its size. In *S. carcini* we identified two genes containing a homeobox that phylogenetically clustered with arthropod *abd-A* genes (Supplementary fig. 1, Supplementary Material online, SACA_007164, SACA_020902) but were located on other scaffolds. These two genes do not have the *abdominal-A* domain that accompanies the homeobox domain of other arthropods. We assume that these copies are probably derived from the original *abd-A* gene. These genes are organized in five exons, thus are not result of retrotransposition.

Because the expression of *abd-A* was not detected in early development of barnacles (Blin et al. 2003; Géant et al. 2006), these copies probably serve other functions. For example, in *Artemia* species, *abd-A* is not expressed in early development but is expressed later in the nervous system (Hsia et al. 2010). Similar neofunctionalization may explain the presence of the two *abd-A-like* genes in *S. carcini*. Immunostaining failed to detect the corresponding protein during early development in *S. carcini* (Blin et al. 2003; Géant et al. 2006), but it is unclear whether the highly derived proteins would have been detected by the antibody.

While abdominal-a and abdominal-b are usually expressed in the abdomen of a developing arthropod embryo, the other gene putatively lost in barnacles, Hox3/zen serves to specify segmental identity along the anteroposterior axis of the arthropod embryo (Hughes and Kaufman 2002). Its reduction in barnacles can be easily linked with their non-segmented body structure. With respect to the genes sex combs reduced (scr) and fushi tarazu (ftz) the situation in barnacles is that *A. amphitrite* seems to have both genes, while in *P. pollicipes* ftz, in *S.carcini* scr is lost. The *S. carcini* homologue of ftz was also named DIVA and is not involved in embryonic patterning, but is expressed in the central nervous system (Mouchel-Vielh et al. 2002). Scr in insects and some crustaceans is involved in development of the appendages of the second maxillary segment and the prothorax (Abzhanov and Kaufman 1999). Nothing is known about its expression pattern in barnacles. Loss of some hox genes is seen in other animal taxa as well, e.g. in nematodes (Aboobaker and Blaxter 2010). Similar to barnacles, other arthropods also lack abd-A and hox3, e.g. mites and pycnogonids (Pace et al. 2016). These reductions often correlate with profound changes in body structure.

### Differential Expression of Genes in *S. Carcini* Tissues

#### Overview of RNA-Seq Data

**Fig. 5.**
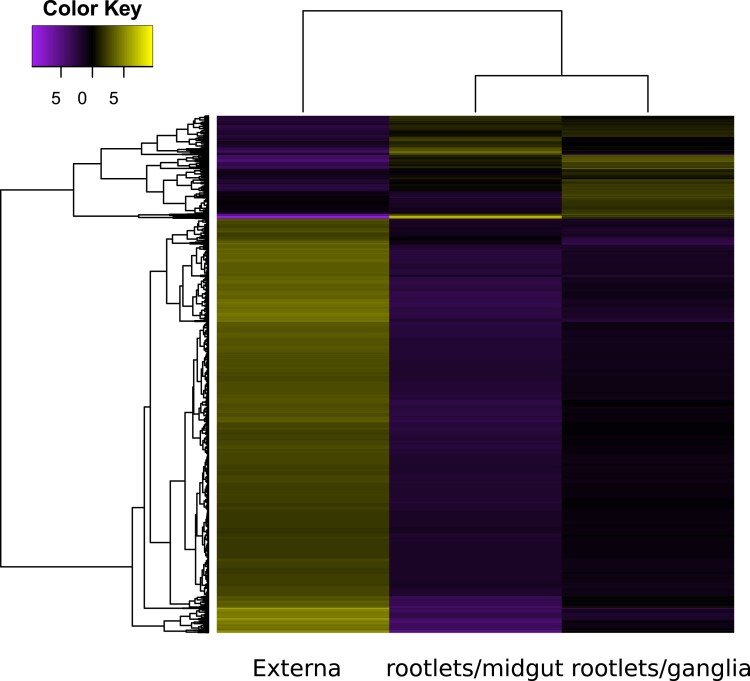
Heat map of transcripts generated from one externa and two interna samples. Only genes with e-values below 1*(E)*-10 (326 genes) were chosen for display. Expression values are plotted in log2 space and mean-centered (mean expression value for each feature is subtracted from each of its expression values in that row). Brightness shows the level of upregulation (right) and downregulation (left).

**Fig. 6.**
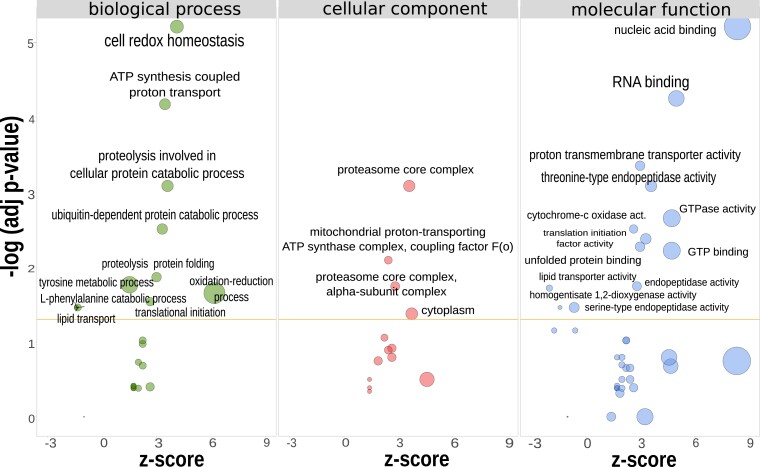
GO term enrichment analysis for DEGs in *S. carcini* interna and externa samples. The z-score is assigned to the x-axis. Negative values correspond to upregulation in the interna, whereas positive values correspond to upregulation in the externa. The y-axis shows the negative logarithm of the adjusted *P*-value (higher values are more likely to be significant). The area of the circles is proportional to the number of genes assigned to the term.

In addition to changes in gene content, the evolution of species is driven by changes in gene expression profiles. We therefore compared RNA-Seq data from the interna (rootlets around the midgut and thoracic ganglia) and externa (breeding sac). This revealed 452 genes that were upregulated specifically in the interna and 1418 genes that were upregulated specifically in the externa (see also Supplementary online material at github.com/smrtin/sacculina_genome_project). Although we identified several commonly upregulated genes in the two samples from the interna, these samples also showed a surprising degree of difference, indicating that the functions of the infiltrating rootlets may differ according to the host region they penetrate, in this case the midgut and thoracic ganglia (fig. 5). Recent studies with other Rhizocephala have shown that rootlets in different regions of the host take on different characteristics (Miroliubov et al. 2020). The 1870 differentially expressed genes (DEGs) included 1012 with conserved protein domains and we were able to assign Gene Ontology (GO) terms to 661 of them. The GOplot (fig. 6) indicates gene expression levels and identified functional groups of genes that are differentially regulated in the interna or the externa.

#### Genes Upregulated in the Interna

GO analysis revealed that several genes upregulated in the interna samples are involved in lipid transport. Many of these genes contain a conserved vitellogenin domain, which serves as a precursor of the lipoproteins and phosphoproteins that make up most of the protein content of yolk. This may indicate that lipids are taken up directly from the host and will probably be transported to the externa, where the eggs are produced. It is notable here that the nauplius larvae are lecitotrophic, thus do not take up food, but live from their yolk supply. Following this hypothesis, there might be more mechanisms for nutrient recycling by an endoparasitic crustacean.

Several other upregulated genes appeared to be involved in amino acid and protein metabolism, including two featuring a homogentisate 1,2-dioxygenase domain with GO-terms related to L-phenylalanine and tyrosine metabolism. The transformation of homogentisate into 4-maleylacetoacetate feeds into the citric acid cycle, suggesting the utilization of host proteins and amino acids as a source of energy, although tyrosine metabolism could also be used to produce the hormones dopamine and adrenaline.

As only 35% of the DEGs could be linked to GO terms, we also characterized genes upregulated in the interna by identifying conserved protein domains using InterPro. This revealed several genes related to developmental processes, including two containing the juvenile hormone binding protein (JHBP) domain, suggesting that the products may regulate the host molting cycle (Qian et al. 2019). We also identified two genes encoding crustacean neurohormones (Chang and Lai 2018). This family of peptides includes crustacean hypoglycemic hormone, which regulates sugar turnover in the hemolymph, molt inhibiting hormone (MIH), which regulates ecdysis, and gonad inhibiting hormone, which regulates gonad development. Although it is unclear whether these substances leave the interna, JHBP and these peptide hormones are clear candidates for the suppression of ecdysis and gonad development in the host as part of the overall strategy of host castration.

Other genes upregulated in the interna encoded putative components of the innate immune system, which may facilitate the infestation of hosts even if they become more susceptible to microbial infection. One such gene, encoding an animal heme peroxidase domain, is likely to be an ortholog of the innate immunity factor peroxidasin in *D. melanogaster* (Parsons and Foley 2016). Another gene showed high sequence similarity to macrophage mannose receptor 1, which carries a lectin domain that binds soluble carbohydrates or carbohydrates that are part of glycoproteins or glycolipids (Sharon and Lis 2004). Among many other functions, lectins are known to play an important role in the innate immune system, acting as pattern recognition receptors by binding to glycoproteins on the surface of microorganisms (Lin et al. 2020).

Finally, we identified an upregulated gene encoding for an otopetrin domain, which allows the formation of proton-selective ion channels. Such channels are an essential component of vertebrate sour taste receptors and are strongly expressed in taste receptor cells (Tu et al. 2018). The presence of otopetrin-domain proteins in the interna may therefore indicate a role in the sampling of nutrients from the host hemolymph, although it is unclear how this influences the parasite-host relationship.

We also observed an unanticipated difference between the two interna samples from the host midgut and thoracic ganglia (fig. 5), supporting recent reports showing the differing morphology and activity of rootlets in the rhizocephalan species *Peltogasterella gracilis* (Miroliubov et al. 2020) and *Sacculina pilosella* (Lianguzova et al. 2021). These studies describe morphological differentiation of those rootlets in contact with host nerve chords and indicate that these sites allow for humoral interactions between the parasite and host.

#### Genes Upregulated in the Externa

The externa features three times as many upregulated genes as the interna, at least partially due to the diversity of tissues in this structure (muscle cells, nerve cells, gonads, epidermis). GO analysis revealed strongly upregulated genes associated with translational initiation and protein folding, which is a characteristic of growing and active tissues. On the other hand, we also found upregulated genes that are involved in ubiquitin-dependent protein catabolism, general proteolysis, and proteolysis involved in cellular protein catabolism. This indicates a high protein turnover, probably related to the development of gonads and earliest larval stages. Another group of upregulated genes was associated with ATP synthesis coupled to proton transport, indicating a strong demand for energy in the externa. This is supported by the upregulation of genes involved in cell redox homeostasis and oxidation-reduction processes. For example, we observed the upregulation of genes encoding thioredoxin, which reduces other proteins by cysteine thiol-disulfide exchange, and glutaredoxin, an oxidation repair enzyme that participates in many cellular functions, including redox signaling and the regulation of glucose metabolism, but also facilitates proper protein folding (Nordberg and Arnér 2001; Berndt et al. 2008). These functions may also be linked to the high protein turnover in developing embryos before the free-swimming larvae leave the externa.

## Conclusions

The draft assembly of the *S. carcini* genome allows the comparison of this endoparasitic barnacle with the sessile barnacles *A. amphitrite* and *P. pollicipes*, as well as with other arthropods. Our initial hypothesis was that genes and gene families may have undergone evolutionary depletion and contraction during the transition to a simplified morphology. However, the number of predicted protein-coding genes in *S. carcini* is in the same range than in the non-parasitic barnacles and most other crustacean genomes. The analysis of ortholog sets revealed a higher percentage of lost genes from gene family clusters that are likely to have been present in the last common ancestor of all arthropods. However, many *S. carcini* genes appear to be unique and do not cluster with orthologs from other arthropods. In addition, a lot of duplication events can also be assumed. This suggests the parasitic lifestyle and morphological streamlining of *S. carcini* led to a high rate of gene turnover in this lineage without triggering extensive gene loss. But due to the differences in gene prediction pipelines of different genome projects and the process of homology predictions these results must be interpreted with caution.

Focusing on the *hox* gene cluster, we combined gene prediction data with mining for orthologs using blast searches of the genomic sequence. We observed some gene losses in this highly conserved hox cluster (*Hox3/zen*, *Scr*, and *abd-A*) correlating with the profound changes in the *S. carcini* body plan (no clear body structure and no appendages in adult *S. carcini*).

In addition to the divergence of the protein repertoire of *S. carcini* from other crustaceans, we also mined differential gene expression data to provide insight into the atypical development and morphology of this species. We observed an unanticipated difference between two interna samples from the host midgut and thoracic ganglia, supporting recent reports showing the differing morphology and activity of rootlets in other rhizocephalan species. Besides various metabolic functions, whose upregulation would be anticipated in a parasitic species, we also observed the expression of immunity-related genes, juvenile hormone binding protein and two crustacean neurohormones (known to suppress ecdysis and gonad development in other species). Future studies should address the direct effect of these humoral factors on host development and immunity. All in all, we conclude that *S. carcini* underwent strong genomic adaptations towards exploting and manipulating its host. Our results are only a first glimpse into the genome biology of this highly aberrant and specialized endoparasite and might also open the field to comparatively study parasitic crustaceans, e.g. from Copepoda or Isopoda.

## Methods

### Sampling Animals and Preparing Tissues

Shore crabs parasitized with *S. carcini* were collected during marine biology excursions to the biological station List/Sylt. Crabs were dredged from a depth of 5–10 m because specimens found on the shore rarely showed signs of infestation. Crabs were visually inspected for parasitic externae and were maintained in seawater aquaria. As *S. carcini* is the only parasitic barnacle occurring here, species determination was not an issue. For nucleic acid isolation, *S. carcini* externae were removed with scissors, and cut into two, three or four pieces. For DNA isolation, the tissue was transferred immediately into 99% molecular biology grade ethanol, and 20–30 mg of tissue was used per DNA isolation procedure. For RNA isolation, the tissue was transferred immediately into RNAlater (Qiagen, Hilden, Germany). RNA was also isolated from internae, which were removed from host crabs on ice, cut into slices and transferred immediately into RNAlater.

### DNA Isolation and Sequencing

DNA was isolated using the Qiagen DNeasy blood and tissue kit, following the recipe for animal tissue, except for using 50 µL (instead of 200 µL as in the standard procedure) of molecular biology grade water for final elution from the silica matrix to yield higher DNA concentration. The DNA quality and amount were checked using a Nanodrop spectrophotometer (Thermo Fisher Scientific, Waltham, MA, USA) and the size and integrity of DNA fragments was evaluated by gel electrophoresis. For long-read sequencing, the DNeasy protocol was modified by replacing the steps where spin columns are used with magnet-based isolation steps, using 0.4 volumes of AMPure magnetic beads (Beckman Coulter, Brea, CA, USA). Short-read sequence data from genomic DNA were produced on Illumina platforms by commercial sequencing services: paired-end libraries (2 × 100 bp) for 500 bp inserts were sequenced with the TrueSeq v3 sequencing chemistry. In addition, mate-pair libraries (Nextera, 2 × 150 bp) were created for 3-kb and 8-kb fragment sizes. Tissue from a single externa was used for Illumina short-read DNA sequencing (see above) and to isolate DNA for long-read sequencing. The DNA isolation procedure as described above yielded 1.5–5 µg of total DNA with each slice of externa tissue. We used 1.5–2.5 µg total DNA for library preparation with a ligation sequencing approach (ONT SQK LSK-109). All in all we carried out five sequencing runs with individual flow cells (v.10.3) on the GridIon platform (ONT Systems), altogether yielding 14.8 Gbp of long-read sequence data.

### RNA Isolation and Sequencing

A different animal was used for transcriptome sequencing. RNA was isolated from three different body parts of an adult *S. carcini* female parasitizing a shore crab: a piece of the external breeding sac; internal rootlets placed around the midgut of the host; and internal rootlets concentrated around the thoracic ganglia of the host. The host was dissected on ice, parasite tissue was placed in RNAlater immediately after dissection and total RNA was isolated using the RNAeasy Kit (Qiagen) for sequencing by Starseq (Mainz, Germany). Sequencing libraries were prepared by Starseq using the TruSeq RNA LT kit (Illumina, San Diego, CA, USA). Insert sizes were selected around 600 bp, and 2 × 300 bp sequencing runs were carried out using the Illumina Miseq platform.

### Trimming and Assembly of Genomic Data

Long reads were basecalled automatically during the sequencing runs with MinKnow (ONT). Porechop (https://github.com/rrwick/Porechop) was used for adaptor trimming. Read lengths below 3 kb were discarded using a custom Perl script. *S. carcini* genome assembly was completed following the evaluation of several long-read assembly tools in parallel: flye v.2.7 (Kolmogorov et al. 2019), miniasm v.0.3 (Li 2016), wtdbg2 (Ruan and Li 2020) and shasta v.0.6.0 (Shafin et al. 2020). Genome assemblies were compared with QUAST (Gurevich et al. 2013) and several parameters (N50, L50, N75, L75, complete assembly size) favored the flye assembly over the others. Final polishing with short Illumina reads (pilon v.1.2.4) (Walker et al. 2014) was carried out to remove the nonrandom sequencing errors frequently found in ONT long-read data. For the definitive version of the draft sequence, initial assembly was therefore carried out using flye v.2.7 with long reads selected for size ≥ 3 kb. The flye assembly underwent subsequent error correction by mapping Illumina short reads with BWA MEM (Li and Durbin 2009) and correcting SNPs and indels with pilon (Walker et al. 2014). These mapping and polishing steps were done twice. Additional scaffolding was achieved using a BWA MEM mapping of Illumina mate pairs (8-kb inserts) and the scaffolding tool BESST (Sahlin et al. 2016).

### Annotation of Repeats and Protein Coding Genes

Repeat content was identified and annotated using Repeatmodeler2 (Flynn et al. 2020) and Repeatmasker. DeviaTE (Weilguny and Kofler 2019) was used to compare repeat content of the assembly and in the raw data. Here artificial reads were generated from the assembly and read coverage for selected repeat families are compared between artificial reads and raw data reads. If there is more coverage found in real sequencing data than in artificial reads aiming to have an even coverage across the assembly, then repeats in the assembly might be underrepresented.

We used funannotate v1.7.4 (https://github.com/nextgenusfs/funannotate) for protein coding gene annotations. We trained the pipeline with RNA-Seq reads that were mapped to the genome. Training involves guided transcriptome assembly with Trinity followed by PASA assembly (Haas et al. 2003, 2008). During the gene prediction step, the *ab initio* predictors Augustus and Genemark are trained, and Evidence Modeler is used to generate consensus gene models from all data. As additional external evidence we used the BUSCO Arthropoda set and provided transcripts from transcriptome assemblies of seven crustacean taxa from NCBI (*Amphibalanus improvisus, Octolasmis warwickii, Glyptelasma gigas, S. balanoides, Lepas anatifera, P. pollicipes, Neolepas marisindica*) and assembled three additional transcriptormes with Trinity based on RNA-Seq data retrieved from NCBI (*Tetraclita japonica formosana, A. amphitrite*, and *P. pollicipes*). A final round of functional annotation using InterPro datasets was carried out using a local installation of interproscan (Jones et al. 2014).

### BUSCO Analysis and Orthologous Gene Clusters

We used BUSCO (v.4) (Seppey et al. 2019) to estimate the completeness of the predicted protein sets. Conserved genes are used as a reference that would be expected in a group of animals. We compared our data with the Arthropoda dataset (derived from orthodb10, with 1013 ortholog sets). We performed an analysis of orthologous protein groups with using orthofinder v2 (Emms and Kelly 2019) adding 17 pancrustacean taxa to our *S. carcini* predicted protein set. We downloaded each genome and the corresponding gene annotation from NCBI and extracted the longest aminoacid sequence per gene. An initial run was only performed with the protein prediction of the 17 taxa and the protein prediction of Sacculina carcini was added later to the existing run. Orthofinder also generated a species tree based on a selection of suitable orthologous sequences. This species tree was re-rooted with the ostracod taxa as suggested by the phylogeny in Schwentner et al. 2018. The orthofinder parts that incorporated this phylogenetic information were rerun. We extracted information on duplication events per node and calculated the number of missing orthogroups per species (fig. 3). Also the number of sequences that were present at the last common ancestor node of the orthogroups was extracted and visualized (fig. 2*A*).

Gene family expansion and contraction was analyzed using orthogroups_count.tsv generated by orthofinder v2 (Emms and Kelly 2019). After filtering the data for extremely large protein families, we used CAFE (v4.2.1) (De Bie et al. 2006; Han et al. 2013) to identify significantly derived values for *S. carcini.* Gene clusters identified as expanded or constricted were further characterized by identifying protein domains from the PFAM-A database, using the hhsearch module from HHsuite3 (Steinegger et al. 2019) for comparison of protein family alignments and all the hmm files of Pfam-A.

To make sure that we did not miss genes in the orthofinder step, we also mined protein sets directly for the homeobox transcription factor domains using HMMsearch from the HMMER package (v3.1.2) (Eddy 2009). For comparison we downloaded all hox domains from *Drosophila melanogaster*, *Tribolium castaneum* and *Apis mellifera* from the homeobox database (Zhong and Holland 2011). We aligned the insect domains with MAFFT v7.3.1 (Katoh and Standley 2014) and added candidate genes from barnacles to this alignment using the local optimization algorithm of MAFFT (linsi) and setting the keeplength and addlong flags. Phylogenetic analysis of the hox domain alignment was then done with FastTree v2.1 (Price et al. 2009).

### Assembly, Annotation and Expression Analysis of Transcriptome Data

Transcriptome short reads were assembled and analyzed using the Trinity package (Grabherr et al. 2011). In order to rule out contamination with host RNA, especially in the case of transcriptomic data from interna tissues, we mapped reads to the genome assembly using bbmap as part of bbtools (BBMap, Bushnell B et al: sourceforge.net/projects/bbmap/) and discarded reads that were not mapped properly to the genome. We generated a transcriptome assembly based on all samples and all read-pairs if at least one read mapped properly to the genome. Functional annotation was carried out using Trinotate (https://github.com/Trinotate), an annotation suite designed for the automatic functional annotation of transcriptomes, particularly *de novo* assembled transcriptomes, from model or non-model organisms (Bryant et al. 2017). Sequences identified as ribosomal RNA were excluded from downstream analysis.

Differential expression was established by estimating transcript abundance across each sample using bowtie2 and RSEM with scripts provided in the Trinity utilities. Differentially expressed transcripts or genes were identified by running the run_DE_analysis.pl-script using the edgeR method (Nikolayeva and Robinson 2014), which performs pairwise comparisons among each sample type. The two samples from the interna were combined, resulting in a comparison between transcripts from the interna and the externa, to extract transcripts that had the strongest differences in expression (most significant FDR and fold-changes) and to cluster the transcripts according to their patterns of differential expression GO annotations. To analyze GO-terms that were differentially expressed in the two tissues, we ran the prep_n_run_GOplot.pl-script to visualize the biological information (Walter et al. 2015).

## Supplementary Material

evac149_Supplementary_Data

## Data Accessibility

All sequencing raw data presented here is accessible via NCBI bioproject number PRJNA659937, with sequence read archive SRR12539549-SRR12539560. Annotations, cds and protein sets, as well as all workflows, scripts and command line parameters are accessible through a github repository, github.com/smrtin/sacculina_genome_project. The bioinformatic analysis steps were combined in a snakemake workflow to document all commands and parameters. A collection of the most important commands can also be found in the Supplementary material online.

## References

[evac149-B1] Aboobaker A , Blaxter M. 2010. The nematode story: hox gene loss and rapid evolution. Adv Exp Med Biol 689:101–110. 20795325 10.1007/978-1-4419-6673-5_7

[evac149-B2] Abzhanov A , Kaufman TC. 1999. Novel regulation of the homeotic gene Scr associated with a crustacean leg-to-maxilliped appendage transformation. Development 126:1121–1128. 10021332 10.1242/dev.126.6.1121

[evac149-B3] Anderson J . 1884. On the genus peltogaster (rathke); an animal form parasitic on the abdomen of crabs. Proc R Phys Soc Edinb 1:412–414.

[evac149-B4] Anderson DT . 1993. Barnacles: structure, function, development and evolution. London: Chapman & Hall.

[evac149-B5] Berndt C , Lillig CH, Holmgren A. 2008. Thioredoxins and glutaredoxins as facilitators of protein folding. Biochim Biophys Acta 1783:641–650. 18331844 10.1016/j.bbamcr.2008.02.003

[evac149-B6] Bernot JP , et al 2022. Chromosome-level genome assembly, annotation, and phylogenomics of the gooseneck barnacle *Pollicipes pollicipes*. GigaScience 11:1–16. 10.1093/gigascience/giac021 PMC8917513 35277961

[evac149-B7] Blin M , Rabet N, Deutsch JS, Mouchel-Vielh E. 2003. Possible implication of hox genes abdominal-B and abdominal-A in the specification of genital and abdominal segments in cirripedes. Dev Genes Evol 213:90–96. 12632178 10.1007/s00427-003-0294-z

[evac149-B8] Bresciani J , Høeg JT. 2001. Comparative ultrastructure of the root system in rhizocephalan barnacles (Crustacea: cirripedia: rhizocephala). J Morphol 249:9–42. 11410937 10.1002/jmor.1039

[evac149-B9] Bryant DM , et al 2017. A tissue-mapped axolotl De Novo transcriptome enables identification of limb regeneration factors. Cell Rep 18:762–776. 28099853 10.1016/j.celrep.2016.12.063 PMC5419050

[evac149-B10] Chang WH , Lai AG. 2018. Comparative genomic analysis of crustacean hyperglycemic hormone (CHH) neuropeptide genes across diverse crustacean species. F1000Res 7:100. 30356453 10.12688/f1000research.13732.1 PMC6178914

[evac149-B11] De Bie T , Cristianini N, Demuth JP, Hahn MW. 2006. CAFE: a computational tool for the study of gene family evolution. Bioinformatics 22:1269–1271. 16543274 10.1093/bioinformatics/btl097

[evac149-B12] Delage Y . 1884. Evolution de la sacculine (sacculina carcini thomps.), crustacé endoparasite de l’ordre nouveau des kentrogonides. Paris: Archives de zoologie expérimentale et générale.

[evac149-B13] Deutsch JS . 2009. Darwin and the cirripedes: insights and dreadful blunders. Integr Zool 4:316–322. 21392304 10.1111/j.1749-4877.2009.00173.x

[evac149-B14] Deutsch JS , Mouchel-Vielh E. 2003. Hox genes and the crustacean body plan. Bioessays 25:878–887. 12938177 10.1002/bies.10319

[evac149-B413] Eddy SR . 2009. A new generation of homology search tools based on probabilistic inference. Genome Inform 23:205–211. 20180275

[evac149-B15] Emms DM , Kelly S. 2019. Orthofinder: phylogenetic orthology inference for comparative genomics. Genome Biol 20:238. 31727128 10.1186/s13059-019-1832-y PMC6857279

[evac149-B16] Flynn JM , et al 2020. Repeatmodeler2 for automated genomic discovery of transposable element families. Proc Natl Acad Sci U S A 117:9451–9457. 32300014 10.1073/pnas.1921046117 PMC7196820

[evac149-B17] Géant E , Mouchel-Vielh E, Coutanceau J-P, Ozouf-Costaz C, Deutsch JS. 2006. Are cirripedia hopeful monsters? Cytogenetic approach and evidence for a hox gene cluster in the cirripede crustacean sacculina carcini. Dev Genes Evol 216:443–449. 16773337 10.1007/s00427-006-0088-1

[evac149-B18] Gibert JM , Mouchel-Vielh E, Quéinnec E, Deutsch JS. 2000. Barnacle duplicate engrailed genes: divergent expression patterns and evidence for a vestigial abdomen. Evol Dev 2:194–202. 11252562 10.1046/j.1525-142x.2000.00059.x

[evac149-B19] Glenner H . 2001. Cypris metamorphosis, injection and earliest internal development of theRrizocephalan loxothylacus panopaei (gissler). Crustacea: cirripedia: rhizocephala: sacculinidae. J Morphol 249:43–75. 11410938 10.1002/jmor.1040

[evac149-B20] Glenner H , Hebsgaard MB. 2006. Phylogeny and evolution of life history strategies of the parasitic barnacles (Crustacea, cirripedia, Rhizocephala). Mol Phylogenet Evol 41:528–538. 16876443 10.1016/j.ympev.2006.06.004

[evac149-B21] Grabherr MG , et al 2011. Full-length transcriptome assembly from RNA-seq data without a reference genome. Nat Biotechnol 29:644–652. 21572440 10.1038/nbt.1883 PMC3571712

[evac149-B22] Gurevich A , Saveliev V, Vyahhi N, Tesler G. 2013. QUAST: quality assessment tool for genome assemblies. Bioinformatics 29:1072–1075. 23422339 10.1093/bioinformatics/btt086 PMC3624806

[evac149-B23] Gurney RH , Grewe PM, Thresher RE. 2006. Mitochondrial DNA haplotype variation in the parasitic cirripede sacculina carcini observed in the cytochrome oxidase gene (COI). J Crust Biol 26:326–330.

[evac149-B24] Haas BJ , et al 2003. Improving the Arabidopsis genome annotation using maximal transcript alignment assemblies. Nucleic Acids Res 31:5654–5666. 14500829 10.1093/nar/gkg770 PMC206470

[evac149-B25] Haas BJ , et al 2008. Automated eukaryotic gene structure annotation using EVidenceModeler and the program to assemble spliced alignments. Genome Biol 9:R7. 18190707 10.1186/gb-2008-9-1-r7 PMC2395244

[evac149-B26] Han MV , Thomas GWC, Lugo-Martinez J, Hahn MW. 2013. Estimating gene gain and loss rates in the presence of error in genome assembly and annotation using CAFE 3. Mol Biol Evol 30:1987–1997. 23709260 10.1093/molbev/mst100

[evac149-B27] Høeg JT . 1995. The biology and life cycle of the Rhizocephala (cirripedia). J Mar Biol Assoc U K 75:517–550.

[evac149-B28] Høeg J , Lutzen J. 1995. Life cycle and reproduction in the cirripedia, Rhizocephala. Oceanogr Mar Biol Annu Rev 33:427–485.

[evac149-B29] Hsia CC , Paré AC, Hannon M, Ronshaugen M, McGinnis W. 2010. Silencing of an abdominal hox gene during early development is correlated with limb development in a crustacean trunk. Evol Dev 12:131–143. 20433454 10.1111/j.1525-142X.2010.00399.x PMC2893884

[evac149-B30] Hughes CL , Kaufman TC. 2002. Hox genes and the evolution of the arthropod body plan. Evol Dev 4:459–499. 12492146 10.1046/j.1525-142x.2002.02034.x

[evac149-B31] Jones P , et al 2014. Interproscan 5: genome-scale protein function classification. Bioinformatics 30:1236–1240. 24451626 10.1093/bioinformatics/btu031 PMC3998142

[evac149-B32] Katoh K , Standley DM. 2014. MAFFT: iterative refinement and additional methods. Methods Mol Biol 1079:131–146. 24170399 10.1007/978-1-62703-646-7_8

[evac149-B33] Kim J-H , et al 2019. Draft genome assembly of a fouling barnacle, *Amphibalanus amphitrite* (darwin, 1854): the first reference genome for thecostraca. Front Ecol Evol 7:465.

[evac149-B34] Kolmogorov M , Yuan J, Lin Y, Pevzner PA. 2019. Assembly of long, error-prone reads using repeat graphs. Nat Biotechnol 37:540–546. 30936562 10.1038/s41587-019-0072-8

[evac149-B35] Kristensen T , et al 2012. The selective advantage of host feminization: a case study of the green crab carcinus maenas and the parasitic barnacle sacculina carcini. Mar Biol 159:2015–2023.

[evac149-B36] Li H . 2016. Minimap and miniasm: fast mapping and de novo assembly for noisy long sequences. Bioinformatics 32:2103–2110. 27153593 10.1093/bioinformatics/btw152 PMC4937194

[evac149-B37] Li H , Durbin R. 2009. Fast and accurate short read alignment with burrows-wheeler transform. Bioinformatics 25:1754–1760. 19451168 10.1093/bioinformatics/btp324 PMC2705234

[evac149-B38] Lianguzova AD , Ilyutkin SA, Korn OM, Miroliubov AA. 2021. Specialised rootlets of sacculina pilosella (Rhizocephala: sacculinidae) used for interactions with its host's Nervous system. Arthropod Struct Dev 60:101009. 33307518 10.1016/j.asd.2020.101009

[evac149-B39] Lin Z , Wang J-L, Cheng Y, Wang J-X, Zou Z. 2020. Pattern recognition receptors from lepidopteran insects and their biological functions. Dev Comp Immunol 108:103688. 32222357 10.1016/j.dci.2020.103688

[evac149-B40] Miroliubov A , et al 2020. Specialized structures on the border between rhizocephalan parasites and their host's Nervous system reveal potential sites for host-parasite interactions. Sci Rep 10:1128. 31980714 10.1038/s41598-020-58175-4 PMC6981121

[evac149-B41] Mouchel-Vielh E , Blin M, Rigolot C, Deutsch JS. 2002. Expression of a homologue of the fushi tarazu (ftz) gene in a cirripede crustacean. Evol Dev 4:76–85. 12004965 10.1046/j.1525-142x.2002.01063.x

[evac149-B42] Mouchel-Vielh E , Rigolot C, Gibert JM, Deutsch JS. 1998. Molecules and the body plan: the hox genes of cirripedes (Crustacea). Mol Phylogenet Evol 9:382–389. 9667986 10.1006/mpev.1998.0498

[evac149-B43] Mouritsen KN , Jensen T. 2006. The effect of sacculina carcini infections on the fouling, burying behaviour and condition of the shore crab. Carcinus maenas. Mar Biol Res 2:270–275.

[evac149-B44] Müller F . 1862. Die rhizocephalen, eine neue gruppe schmarotzender kruster. Archive für Naturgeschichte 1:1–9.

[evac149-B45] Nikolayeva O , Robinson MD. 2014. Edger for differential RNA-seq and ChIP-seq analysis: an application to stem cell biology. Methods Mol Biol 1150:45–79. 24743990 10.1007/978-1-4939-0512-6_3

[evac149-B46] Nordberg J , Arnér ES. 2001. Reactive oxygen species, antioxidants, and the mammalian thioredoxin system. Free Radic Biol Med 31:1287–1312. 11728801 10.1016/s0891-5849(01)00724-9

[evac149-B47] Nunez JCB , et al 2021. Ecological load and balancing selection in circumboreal barnacles. Mol Biol Evol 38:676–685. 32898261 10.1093/molbev/msaa227 PMC7826171

[evac149-B48] Pace RM , Grbic M, Nagy LM. 2016. Composition and genomics organisation of arthropod hox clusters. EvoDevo 7:11. 27168931 10.1186/s13227-016-0048-4 PMC4862073

[evac149-B49] Parsons B , Foley E. 2016. Cellular immune defenses of drosophila melanogaster. Dev Comp Immunol 58:95–101. 26748247 10.1016/j.dci.2015.12.019

[evac149-B50] Pérez-Losada M , et al 2008. The tempo and mode of barnacle evolution. Mol Phylogenet Evol 46:328–346. 18032070 10.1016/j.ympev.2007.10.004

[evac149-B51] Pérez-Losada M , Høeg JT, Crandall KA. 2004. Unraveling the evolutionary radiation of the thoracican barnacles using molecular and morphological evidence: a comparison of several divergence time estimation approaches. Syst Biol 53:244–264. 15205051 10.1080/10635150490423458

[evac149-B52] Powell A , Rowley AF. 2008. Tissue changes in the shore crab carcinus maenas as a result of infection by the parasitic barnacle sacculina carcini. Dis Aquat Organ 80:75–79. 18714687 10.3354/dao01930

[evac149-B53] Price MN , Dehal PS, Arkin AP. 2009. Fasttree: computing large Minimum evolution trees with profiles instead of a distance matrix. Mol Biol Evol 26:1641–1650. 19377059 10.1093/molbev/msp077 PMC2693737

[evac149-B54] Qian H , et al 2019. Cloning and expression analysis of a cytoplasmic juvenile hormone-binding protein from the mud crab, Scylla paramamosain (decapoda, Brachyura, portunidae). Crustaceana 92:907–919.

[evac149-B55] Queinnec E , et al 1999. Cloning and expression of the engrailed.a gene of the barnacle sacculina carcini. Dev Genes Evol 209:180–185. 10079361 10.1007/s004270050242

[evac149-B56] Rabet N , Gibert JM, Quéinnec E, Deutsch JS, Mouchel-Vielh E. 2001. The caudal gene of the barnacle sacculina carcini is not expressed in its vestigial abdomen. Dev Genes Evol 211:172–178. 11455431 10.1007/s004270100142

[evac149-B57] Rasmussen E . 1959. Behaviour of sacculinized shore crabs (carcinus maenas pennant). Nature 183:479–480.

[evac149-B58] Regier JC , et al 2010. Arthropod relationships revealed by phylogenomic analysis of nuclear protein-coding sequences. Nature 463:1079–1083. 20147900 10.1038/nature08742

[evac149-B59] Rowley AF , et al 2020. Prevalence and histopathology of the parasitic barnacle, sacculina carcini in shore crabs, carcinus maenas. J Invertebr Pathol 171:107338. 32035933 10.1016/j.jip.2020.107338

[evac149-B60] Ruan J , Li H. 2020. Fast and accurate long-read assembly with wtdbg2. Nat Methods 17:155–158. 31819265 10.1038/s41592-019-0669-3 PMC7004874

[evac149-B61] Sahlin K , Chikhi R, Arvestad L. 2016. Assembly scaffolding with PE-contaminated mate-pair libraries. Bioinformatics 32:1925–1932. 27153683 10.1093/bioinformatics/btw064

[evac149-B62] Scholtz G , Ponomarenko E, Wolff C. 2009. Cirripede cleavage patterns and the origin of the Rhizocephala (Crustacea: thecostraca). Arthropod Syst Phylogeny 67:219–228.

[evac149-B63] Schwentner M , Richter S, Rogers DC, Giribet G. 2018. Tetraconatan phylogeny with special focus on malacostraca and branchiopoda: highlighting the strength of taxon-specific matrices in phylogenomics. Proc Biol Sci 285:20181524. 10.1098/rspb.2018.1524 PMC6125901 30135168

[evac149-B64] Seppey M , Manni M, Zdobnov EM. 2019. BUSCO: assessing genome assembly and annotation completeness. Methods Mol Biol 1962:227–245. 31020564 10.1007/978-1-4939-9173-0_14

[evac149-B65] Shafin K , et al 2020. Nanopore sequencing and the shasta toolkit enable efficient de novo assembly of eleven human genomes. Nat Biotechnol 38:1044–1053. 32686750 10.1038/s41587-020-0503-6 PMC7483855

[evac149-B66] Sharon N , Lis H. 2004. History of lectins: from hemagglutinins to biological recognition molecules. Glycobiology 14:53R–62R. 10.1093/glycob/cwh122 15229195

[evac149-B67] Steinegger M , et al 2019. HH-suite3 for fast remote homology detection and deep protein annotation. BMC Bioinf 20:473. 10.1186/s12859-019-3019-7 PMC6744700 31521110

[evac149-B68] Thompson JV . 1830. Memoir IV. On the cirripedes, or barnacles; demonstrating their deceptive character; the extraordinary metamorphosis they undergo, and the class of animals to which they indisputably belong. Zool Res 3:69–82.

[evac149-B69] Tu Y-H , et al 2018. An evolutionarily conserved gene family encodes proton-selective ion channels. Science 359:1047–1050. 29371428 10.1126/science.aao3264 PMC5845439

[evac149-B70] Vakirlis N , Carvunis A-R, McLysaght A. 2020. Synteny-based analyses indicate that sequence divergence is not the main source of orphan genes. eLife 9:e53500. 32066524 10.7554/eLife.53500 PMC7028367

[evac149-B71] Walker G . 2001. Introduction to the Rhizocephala (Crustacea:cirripedia). J Morphol 249:1–8. 11410936 10.1002/jmor.1038

[evac149-B72] Walker BJ , et al 2014. Pilon: an integrated tool for comprehensive microbial variant detection and genome assembly improvement. PLoS One 9:e112963. 25409509 10.1371/journal.pone.0112963 PMC4237348

[evac149-B73] Walter W , Sánchez-Cabo F, Ricote M. 2015. GOplot: an R package for visually combining expression data with functional analysis. Bioinformatics 31:2912–2914. 25964631 10.1093/bioinformatics/btv300

[evac149-B74] Weilguny L , Kofler R. 2019. DeviaTE: assembly-free analysis and visualization of mobile genetic element composition. Mol Ecol Resour 19:1346–1354. 31056858 10.1111/1755-0998.13030 PMC6791034

[evac149-B75] Weisman CM , Murray AW, Eddy SR. 2020. Many, but not all, lineage-specific genes can be explained by homology detection failure. PLoS Biol 18:e3000862. 33137085 10.1371/journal.pbio.3000862 PMC7660931

[evac149-B76] Zhong YF , Holland PWH. 2011. HomeoDB2: functional expansion of a comparative homeobox gene database for evolutionary developmental biology. Evol Develop 13:567–568. 10.1111/j.1525-142X.2011.00513.x PMC3399086 23016940

