# Correction to: Genomic Adaptations to an Endoparasitic Lifestyle in the Morphologically Atypical Crustacean *Sacculina carcini* (Cirripedia: Rhizocephala)

**DOI:** 10.1093/gbe/evac179

**Published:** 2022-12-26

This is a correction to: Sebastian Martin, Peter Lesny, Henrik Glenner, Jochen Hecht, Andreas Vilcinskas, Thomas Bartolomaeus, Lars Podsiadlowski, Genomic Adaptations to an Endoparasitic Lifestyle in the Morphologically Atypical Crustacean *Sacculina carcini* (Cirripedia: Rhizocephala), Genome Biology and Evolution, Volume 14, Issue 10, October 2022, evac149, https://doi.org/10.1093/gbe/evac149

In the originally published version of this manuscript, the Data Accessibility section contained an error, whereby the number “PRJNA659937” was incorrectly given as “PRNJA659937”.

This error has been corrected.

