# Supplementary material for: Genomic Adaptations to an Endoparasitic Lifestyle in the Morphologically Atypical Crustacean Sacculina carcini (Cirripedia: Rhizocephala)
Source: Genome Biol Evol. 2022 Oct 12;14(10):evac149. doi: 10.1093/gbe/evac149 (PMC9582164; doi:10.1093/gbe/evac149)
Supplement: evac149_Supplementary_Data [file evac149_supplementary_data.pdf]

Supplementary Material

p. 2 Overview of analysis steps and commands

p. 5 Collection of command lines and parametrs

p. 12 Genome sequencing, assembly and quality check

p. 14 Protein sequences of S.carcini hox genes

p. 16 Protein sequences of S.carcini otopetrin

p. 17 Protein sequences of S.carcini Crustacean neurohormones and JHBP

p. 18 Suppl. figure 1: tree of hox gene domains (barnacles and insects)

p. 19 Suppl. table 1: Comparison of barnacle genome metrics

p. 20 Suppl. table 2: genome assemblies used in the comparative analyses.

p. 21 Suppl. table 3: Gene families that underwent expansions in *S. carcini*

p. 26 Suppl. table 4: Gene families that underwent contractions and reductions in *S. carcini*

.

1. Overview of analysis steps

Analysis pipeline for genome assembly

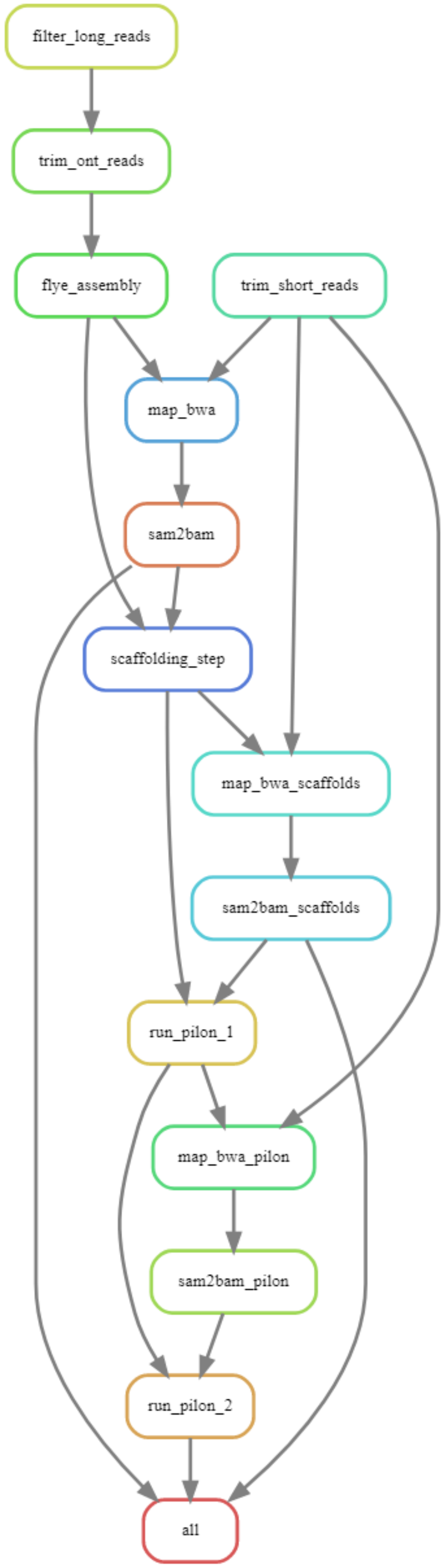

Analysis pipeline for genome annotation

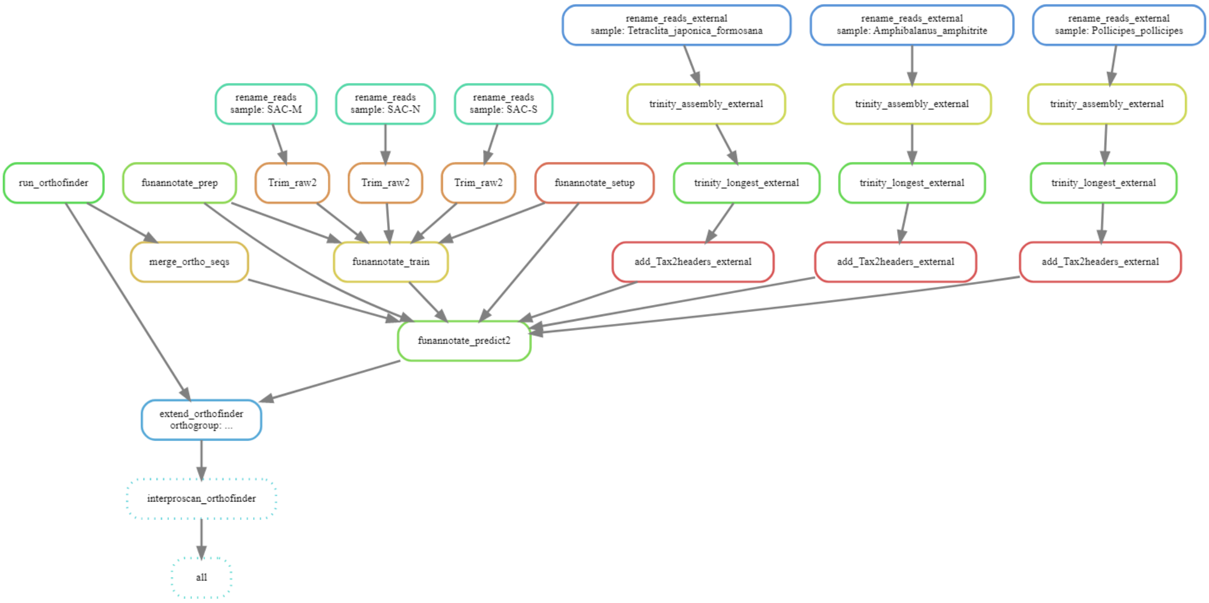
rename_reads_external
sample: Tetraclita_japonica_formosana
rename_reads_external
sample: Amphibalanus_amphitrite
rename_reads_external
sample: Pollicipes_pollicipes
rename_reads
sample: SAC-M
rename_reads
sample: SAC-N
rename_reads
sample: SAC-S
trinity_assembly_external
trinity_assembly_external
trinity_assembly_external
run_orthofinder
funannotate_prep
Trim_raw2
Trim_raw2
Trim_raw2
funannotate_setup
trinity_longest_external
trinity_longest_external
trinity_longest_external
merge_ortho_seqs
funannotate_train
add_Tax2headers_external
add_Tax2headers_external
add_Tax2headers_external
funannotate_predict2
extend_orthofinder
orthogroup: ...
interproscan_orthofinder
all

Analysis pipeline for trancriptome differential expression

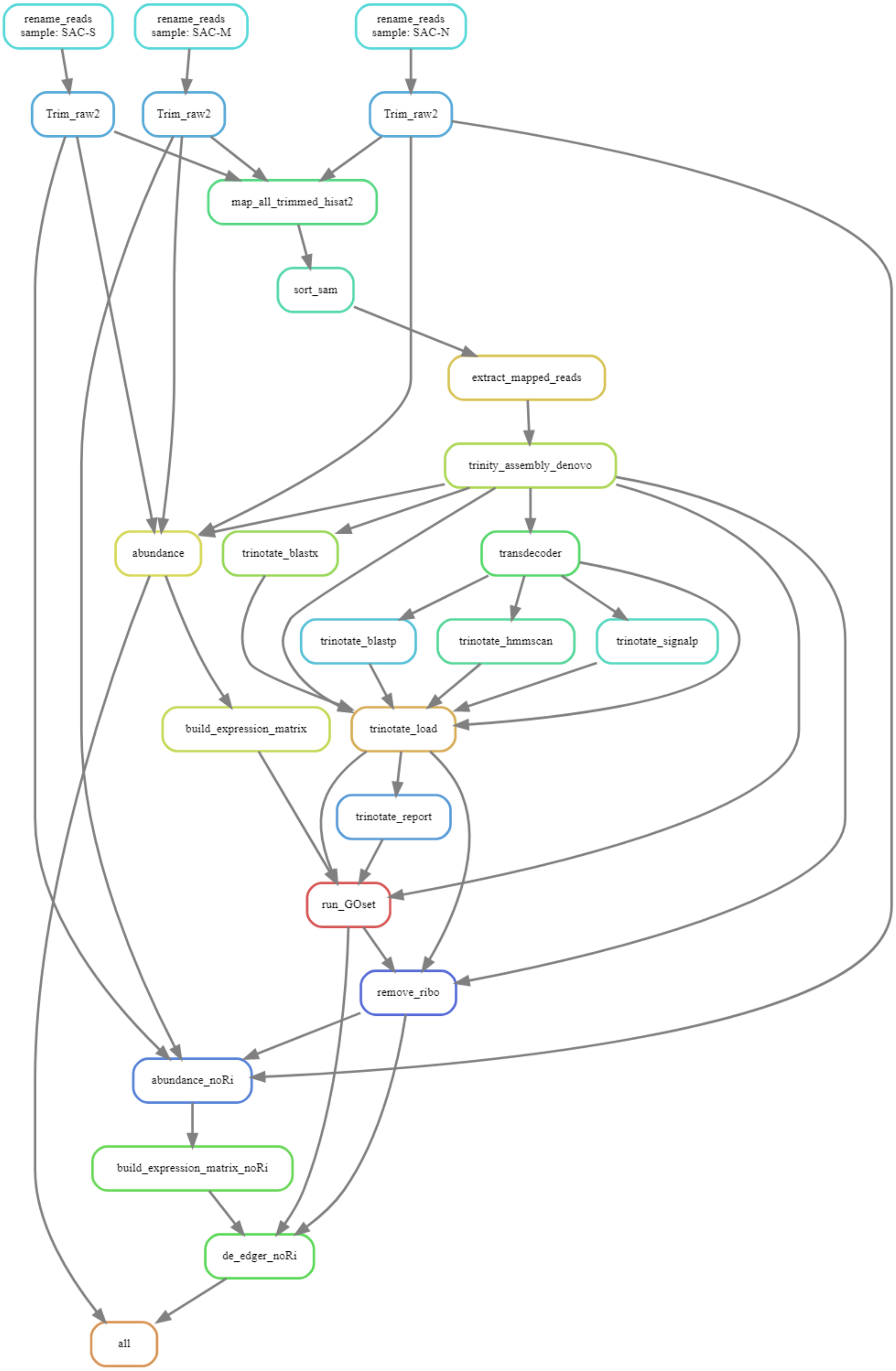

Collection of command lines and parameters

For a more detailed description, please refer to

https://github.com/smrtin/sacculina_genome_project/

(a) Trimming and assembly of genomic data

filter long reads:

perl workflow/scripts/filterseqlength.pl {input} 3000 1000000 {output}

trim ont reads:

porechop -t {threads} --discard_middle -i {input} -o {output}

flye assembly:

flye --nano-raw {input} --genome-size 400m --threads {threads} --out-dir {params.out_dir}

trim short reads:

fastp -i {input.R1} -I {input.R2} -o {output.R1} -O {output.R2} --length_required 100 --low_complexity_filter --detect_adapter_for_pe --trim_poly_x --thread {threads} --json results/genome_assembly/trim_short/saca.fastp.json --html results/genome_assembly/trim_short/saca.fastp.html

map bwa:

bwa index -p {params.prefix} -a is {input.genome}

bwa mem -o {output} -t {threads} {params.prefix} {input.fwd} {input.rev}

sam to bam:

samtools view -Sb {input} > {output.bam}

samtools sort {output.bam} > {output.sort}

samtools index {output.sort}

scaffolding step:

runBESST --no_score -m {params.insert_size} -s {params.std_dev} -c {input.genome} -f {input.bam} -o {params.out_dir} -orientation rf

run pilon:

export _JAVA_OPTIONS='-Xmx500g' ;mkdir -p {params.prefix}

pilon --genome {input.fasta} --fix snps,indels,gaps --changes --frags {input.bam} --output {output}

(b) Annotation of repeats and protein coding genes

repeat modeler:

BuildDatabase -engine ncbi -name MyDatabase Genome.fasta

RepeatModeler -database MyDatabase -engine ncbi -pa {threads}

repeatmasker:

RepeatMasker Genome.fasta -lib ../../../{input} -pa {threads}

calcDivergenceFromAlign.pl -s Assembly_div_summary Genome.fasta.cat.gz

createRepeatLandscape.pl -div Assembly_div_summary -g 365000000 > Genome_repeatlandscape.html

Trim 8kb:

fastp -i {input.R1} -I {input.R2} -o {output.R1} -O {output.R2} --length_required 100 --thread {threads} --low_complexity_filter --detect_adapter_for_pe --trim_poly_x --json results/logs/fastp/{wildcards.sample}.fastp.json --html results/logs/fastp/{wildcards.sample}.fastp.html

Trinity assembly external:

Trinity --seqType fq --left {input.R1} --right {input.R2} --CPU {threads} --SS_lib_type FR --no_version_check --max_memory {params.max_ram} --output {params.outdir} --full_cleanup

sleep 100 ; cp {params.outdir}.Trinity.fasta {output.out}

trinity longest external:

$TRINITY_HOME/util/misc/get_longest_isoform_seq_per_trinity_gene.pl {input} > {output}

funannotate setup:

funannotate setup --install {params.databases} --busco_db {params.busco} --database {params.DB}

funannotate preparation:

funannotate clean --input {input.Genome} --out {output.clean} --pident 95 --cov 95 --minlen 500

funannotate sort --input {output.clean} --out {output.sort}

funannotate train:

funannotate train --input {input.Genome} --cpus {threads} --species "{params.spec}" --max_intronlen {params.max_intron} --out {params.out_dir} --left {input.R1} --right {input.R2}

funannotate predict:

funannotate predict --input {input.Genome} --transcript_evidence {input.TSA} {input.Cirri_assembled} --protein_evidence {input.orthoProt} --cpus {threads} --species "{params.spec}" --max_intronlen {params.max_intron} --out {params.out_dir} --optimize_augustus --organism other --busco_db arthropoda --busco_seed_species fly --name SACA --keep_evm

interproscan funannotate:

interproscan.sh -input {input} -formats xml -disable-precalc --goterms --outfile {output} --seqtype p --tempdir {params.tmp} --verbose --cpu {threads} --applications {params.apps}

funannotate annotate:

funannotate annotate --input {params.predict_dir} --gff {input.gff} --fasta {input.genome} --iprscan {input.iprscan} --busco_db {params.busco} --out {params.out_dir} --cpus {threads}

(c) BUSCO analysis and comparison to other protein sets

busco genome:

busco -f -m {params.mode} -i {input} -o {params.dir} -l {params.lineage} --cpu {threads} --config {params.config} &> {log}

busco predicted proteins:

busco -f -m {params.mode} -i {input} -o {params.dir} -l {params.lineage} --cpu {threads} --config {params.config}

run orthofinder:

orthofinder -M msa -t {threads} -f {params.input} -y -o {params.base} -n {params.postfix}

extend orthofinder:

orthofinder -M msa -t {threads} -b {params.wrkdr} -f {params.input} -y -n {params.postfix}

rerun orthofinder rerooted tree:

echo {params.outgroups} | gotree reroot outgroup -i results/orthofinder/run_1/Results_my_output/WorkingDirectory/OrthoFinder/Results_my_output2/Species_Tree/SpeciesTree_rooted.txt -l - > results/orthofinder/SpeciesTree_rooted.nwk

orthofinder -M msa -t {threads} -ft results/orthofinder/run_1/Results_my_output/WorkingDirectory/OrthoFinder/ Results_my_output2 -y -s results/orthofinder/SpeciesTree_rooted.nwk -n {params.postfix}

interproscan orthofinder:

interproscan.sh -input {input} -formats tsv -disable-precalc --goterms --outfile {output} --seqtype p --tempdir {params.tmp} --verbose --cpu {threads} --applications {params.apps}

(d) Assembly, annotation and expression analysis of transcriptome data

Trim RNA-Seq:

fastp -i {input.R1} -I {input.R2} -o {output.R1} -O {output.R2} --length_required 100 --thread {threads} --low_complexity_filter --detect_adapter_for_pe --trim_poly_x --json results/logs/fastp/{wildcards.sample}.fastp.json --html results/logs/fastp/{wildcards.sample}.fastp.html

map_all_trimmed_hisat2:

hisat2-build -p {threads} {input.Genome} {params.idx} ; hisat2 -S {output} --no-softclip --threads {threads} --dta -x {params.idx} --max-intronlen 300000 --pen-noncansplice 1000000 -1 {fwd_list} -2 {rev_list}

sort sam:

samtools view -@ {threads} -Sb {input} > {output.bam}

samtools sort -@ {threads} {output.bam} > {output.sort}

extract mapped reads:

bedtools bamtofastq -i {input} -fq {output.R1} -fq2 {output.R2} &> {log}

trinity assembly denovo:

Trinity --seqType fq --left {input.R1} --right {input.R2} --SS_lib_type FR --NO_SEQTK --CPU {threads} --max_memory {params.max_ram} --output {params.outdir}

transdecoder:

TransDecoder.LongOrfs -t {input} -m {params.min_prot_length} --G universal --output_dir {params.out_dir}

trinotate_blastx:

blastx -query {input} -db {params.DB} -num_threads {threads} -max_target_seqs 1 -outfmt 6 -evalue 1e-3 > {output}

trinotate_blastp:

blastp -query {input} -db {params.DB} -num_threads {threads} -max_target_seqs 1 -outfmt 6 -evalue 1e-3 > {output}

trinotate_hmmscan:

hmmscan --cpu {threads} --domtblout {output} {params.DB} {input} &> {log}

trinotate_signalp:

```
pyfasta split -n {threads} {output.pep}


for i in $(seq 0 $( expr {threads} - 1 )) ;do
  signalp -f short -v -T {output.dir} -n {output.dir}/signalp_tmp$i.out {output.pep}.$i &
done
wait
cat {output.dir}/signalp_tmp* > {output.final}
```

trinotate_load:

```
$TRINITY_HOME/util/support_scripts/get_Trinity_gene_to_trans_map.pl {input.trans} > {output.map}
cp {params.sqlite} {output.db}
cd {params.work_dir}
Trinotate $(basename {output.db}) init --gene_trans_map $(basename {output.map}) --transcript_fasta ../../{input.trans} --transdecoder_pep ../../{input.pep}
Trinotate $(basename {output.db}) LOAD_swissprot_blastp $(basename {input.blastp})
Trinotate $(basename {output.db}) LOAD_swissprot_blastx $(basename {input.blastx})
Trinotate $(basename {output.db}) LOAD_pfam $(basename {input.hmmscan})
Trinotate $(basename {output.db}) LOAD_signalp $(basename {input.signalp})
```

trinotate_report:

Trinotate {input.db} report > {output.report}

run_GOset:

extract_GO_assignments_from_Trinotate_xls.pl --Trinotate_xls {input.trinotate_report} -G --include_ancestral_terms > {output.go_annot}

$TRINITY_HOME/util/misc/fasta_seq_length.pl {input.trans} > {output.seq_len}

$TRINITY_HOME/util/misc/TPM_weighted_gene_length.py --gene_trans_map {input.map} --trans_lengths {output.seq_len} --TPM_matrix {input.iso_matrix} > {output.gene_len}

add_functional_annotation_to_exprMatrix:

Trinotate_get_feature_name_encoding_attributes.pl {input.report} > {output.mapping}

$TRINITY_HOME/Analysis/DifferentialExpression/rename_matrix_feature_identifiers.pl {input.iso_matrix} {output.mapping} > {output.annot}

estimate abundance:

$TRINITY_HOME/util/align_and_estimate_abundance.pl --transcripts assembly.fas --seqType fq \

--samples_file samplefile.txt --est_method RSEM --aln_method bowtie2 --trinity_mode \

--prep_reference --thread_count {threads} --coordsort_bam

build_expression_matrix:

$TRINITY_HOME/util/abundance_estimates_to_matrix.pl --est_method RSEM \

--gene_trans_map assembly.fas.gene_trans_map \

--name_sample_by_basedir $(cut -f2 samplefile.txt | sed 's|$|/RSEM.isoforms.results|')

de_edger:

$TRINITY_HOME/Analysis/DifferentialExpression/run_DE_analysis.pl --matrix {input.gene_matrix} --method edgeR --samples_file {params.samples} --output {output.dir} --dispersion {params.dispersion}

cd {output.dir}

$TRINITY_HOME/Analysis/DifferentialExpression/analyze_diff_expr.pl --matrix RSEM.gene.TMM.EXPR.matrix.*.count_matrix -P 1e-10 -C 2 --examine_GO_enrichment --GO_annots ../../{input.go_annot} --gene_lengths ../../{input.gene_len}

cat *UP.subset.GOseq.enriched | sort | uniq > all-UP.enrich

$TRINITY_HOME/Analysis/DifferentialExpression/prep_n_run_GOplot.pl --GO_annots ../../{input.go_annot} --DE_subset

RSEM.gene.TMM.EXPR.matrix.externa_vs_interna.edgeR.DE_results.P1e-10_C2.DE.subset --DE_GO_enriched all-UP.enrich \

--tmpdir {params.tmpdir} --pdf_filename {params.plot}

remove ribosomal sequences:

```
grep -B1 -P '^name:.*ribosom.*' resources/funannotate_db/go.obo  | grep '^id' | cut -f2,3 -d':' | sed 's/^ //' > {output.Ribo_GO}
grep -f {output.Ribo_GO} {input.go_annot} | cut -f1 > {output.seq_list_Ribo}
grep -v -f {output.Ribo_GO} {input.go_annot} | cut -f1 > {output.seq_list_noRi}
 grep -f {output.seq_list_Ribo} {input.map} | cut -f2 > {output.seq_withRibo}
grep -v -f {output.seq_list_Ribo} {input.map}  > {output.map_noRi}
seqkit grep -v -f {output.seq_withRibo} {input.assembly} > {output.assembly}
grep -v -f {output.seq_list_Ribo} {input.gene_len} > {output.gene_len}
```

get_trinity_protein_pairs:

```
makeblastdb -in {input.prot} -dbtype prot -title protein_DB -out results/DE_noRibo/protein_DB
blastx -query {input.assembly} -db results/DE_noRibo/protein_DB -max_target_seqs 1 -num_threads {threads} -outfmt 7 -out results/DE_noRibo/blast_output.txt
cat results/DE_noRibo/blast_output.txt |awk '/hits found/{{getline;print}}' | grep -v "#" > {output}
```

2. Genome sequencing, assembly and quality check

We used a combination of short-read (Illumina) and long-read (ONT) data for genome sequencing. This generated a final yield (only reads > 1 kb) of 5.5 million reads covering 14.84 Gb (read N50 = 3199 bp). Although a published source lists 670 Mb as the genome size for *S. carcini* (Animal genome size database, entry based on Rheinsmith et al. 1974), kmer-based and coverage-based analysis of our sequence data suggest a genome size of 450–500 Mb. This suggests our long-read data achieved 30x genome coverage with good quality reads. Our short-read data generated a yield of ~39 Gb (about 80x genome coverage) of paired-end reads (2x 150 bp from 500–600 bp inserts), ~16 Gb of 8-kb insert mate-pairs, and 17 Gb of 3-kb 150-bp mate-pairs (together corresponding to ~65x genome coverage).

Although long reads were used for the initial assembly, short reads were used for polishing SNPs, indels and small gaps. ONT assemblies frequently have non-random errors, especially in homopolymer regions. Finally, 8-kb mate-pair short reads were used to further improve scaffolding. The final assembly was 298.35 Mb in total, comprising 13,055 scaffolds (0.4% Ns) built from 13,532 contigs (scaffold N50 = 161 kb, contig N50 = 124 kb). Therefore, our assembly quality is between the two published cirriped assemblies available: it is more fragmented than the *Amphibalanus amphitrite* genome (Kim et al 2019), which has 2,240 scaffolds (3,312 contigs) with a N50 values of of 458 kb (scaffolds) and 313 kb (contigs); the recently published genome assembly of *Semibalanus balanoides* (Nunez et al. 2021) is more fragmented than the *S.carcini* assembly, with 16,596 scaffolds (18,316 contigs) and N50 values of 56,748 (scaffolds) and 45,551 (contigs) (suppl. Table 1).

While our assembly is significantly smaller than the predicted genome size, several results let us assume that our recent assembly covers most of the non-redundant genetic information: (a) analysis of the genome with the BUSCO reference dataset for arthropods resulted in 75.5% of complete single-copy genes and 13.2% complete and duplicated genes. Fragmented BUSCO genes were 4% and only 7% of the reference genes were missing in the genome. Although this result builds on a very simple and therefore unreliable gene prediction using AUGUSTUS (Stanke & Morgenstern 2005) it hints to a good representation of the whole genome by our assembly (see also BUSCO results using the predicted protein sets); (b) mapping of genomic Illumina short reads (bwa-mem) yielded 94% success rate for single-read mapping (90% properly paired); (c) mapping transcriptome reads from the externa (internal tissue probably contains a substantial amount of RNA from host tissue) also has a high success rate, with more than 98% of reads mapped to the genome assembly. Thus, the discrepancy in size between the estimate and our assembly may reflect the

presence of redundant genome components, such as low-complexity regions as well as simple and complex repeats that are not completely covered by our assembly.

References

Kim JH, Kim HK, Kim H, Chan BKK, Kang S, Kim W. Draft genome of a fouling barnacle, Amphibalanus amphitrite (Darwin, 1854): the first reference genome for Thecostraca. Frot Ecol Evol 2019; 7: 465 doi: 10.3389/fevo.2019.00465

Nunez JCB, Rong S, Damian-Serrano A, Burley JT, Elyanow RG, Ferranti DA, Neil KB, Glenner H, Rosenblad MA, Blomberg A, Johannesson K, Rand DM. Ecological Load and Balancing Selection in Circumboreal Barnacles. Mol Biol Evol. 2021; 38: 676-685.

Rheinsmith EL, Hinegardner R, Bachmann K. Nuclear DNA amounts in crustacea. Comp Biochem Physiol B. 1974; 48: 343-8. doi: 10.1016/0305-0491(74)90269-7

Stanke M, Morgenstern B. AUGUSTUS: a web server for gene prediction in eukaryotes that allows user-defined constraints. Nucleic Acids Res. 2005; 33: W465-7. doi: 10.1093/nar/gki458.

Sequences of *Sacculina carcini* hox genes

>SACA_001202-T1 Abd-B
MAPYHEHFECCSSHFAKNPGAATALYFIGRLYRKTFATHYENFTSLKYYEECANKGVLVALSEGGFYL
TSVRAVRSNSLLSAGRRFNNDSTRFNNDSTRDHDYRSQLILNLQHVRAAVITTPHCSLAVPMNATAVH
SSTTSGVGAGGYDTSMRVPPLQPSPSTSLHHAVPSMATNSTMAKRAALGYSDEAAWHYGGVTDALL
PTSVTAASTVTAFDGGGYPAVAAAYYERKPQPAACWSGKLDYGPPQEPAAPSVAVGCPAFGALPAS
ASTVTMAPAASGAATGAAVWTSGGYVGYGRVPAAFDPVSGQPLPYLTSGDPTYAAAAANQAAVAGL
HHDPYSLRTHGYPGVEALQSVGYHSGHLSSNPLEWTGNVSVRKKRKPYSKYQTLELEKEFLFNAYVS
KQKRWELARNLNLTERQVKIWFQNRRMKNKKAQQRSQPPQEPGTSVGNGKSV

>SACA_001207-T1 Ubx
MTDNNIALRPYFEQGGFYQRSTPADQSGYPVAAAAAGFLSGFPSYGQTRNGVPTGAGGTTNGQESP
FSDAASVAAANACKLYQNGDVGVGTTNGYKLDCAAAAAAVAAKDAVASGYSPKELTNWAGSAAAAG
RFAGLGGAATAHGASVMETAAAVRDRQAAAASNWLAACNQASPGGGGGVGGQTQAAAAHLMQQP
AAQYTSTPIYPWMAIAARRFCLMRATNGSKSAWQNGAVKLA

>SACA_001213-T1 Antp
MPNMTFGNGTGISSSGGAGSTYQMPACSQEMVQSDYSAVVDSSTAHCDPTALRSAYYSGLSGQPM
TAADVNVPVAGTTTATGEHLPSDLHEQQHRATDMYRSYMRPNGVHSQQQQQQMHQQQQQHCQAY
YGQQPPSLELSDPYETGIAAPLDQSQFRAQSPSAQYGAPPPPHLQTVQHHGHHQMTHPHLQHQTLQ
HPQQQQQQQRYMSACKMHDAMAMSNGAVALSDAVYMQTPCEAPDMIGAAGQTAVMYGGQATSAP
GVLNAQQQPPSQAGSPNVLFPWMRTQFDRKRGRQTYTRYQTLELEKEFHFNRYLTRRRRIEIAHALC
LTERQIKIWFQNRRMKWKKENKAKDGSVKSGSRSENETSPGDP

>SACA_001216-T1 ftz
MSTWNLSAGGTAQETHDNWSQQLQQYHQHQLHANSFYQQYHQHHSQQQQQQQQALACEPSTLP
SQLYAHHGAKYSDYHSAAVTTSGMQHYHPTSRYHPSTTGSTFQHHHTGLPGQQQLQQHQVDSVTE
AAGIANSDGQTPTAEEQRTYRMAKMTAAAAAAFTMGALPDQLMTTPSFDVHGQLPPTPTSSTEDIHN
LSLPVKSAAHAIFPWMKVPYAVDPCVTAKRTRQSYTRYQTLELEKEFRTNRYLTRRRRIEIARTVALTE
RQIKIWFQNRRMKAKKEHKPADSSGQTEESFSSEPSLSSSSLHTASSSASVCDVKECATSSCVTTCS
RNIPAADYQPSFVSAGSLPTSMEPSRDPYRHHQHHHLVTAGPGLLAPGYTPMSSVKCEMAI

>SACA_001217-T1 Dfd
MNSFLMNSAAYVDPKFPPSEEYSQASYIPHADYYHQQHMPYGYPQAVSLGYGRDAGSYSSGMAAA
AAAAAYYNQHQPFVAAAPTHQPSPAASLQQLHHQQLPHHVPQQLAVSSGGCSTTMRHQLLHSPPPA
SHHHQPQPPHALPAAGGVVGHQLQLLTAAEQLPASDKCDEDDDEDVDDEDRTPEETVDAADSTGRV
IYPWMKKIHVAGAGEWIKNNGAFQPGMEPKRQRTAYTRHQILELEKEFHFNRYLTRRRRIEIAHSLCLT
ERQIKIWFQNRRMKWKKDNKLPNTKNVRRKTNPAGVTTVVAAKSSSSTASSAASAASATISTAAGCV
SVSAQQQRTAATTTVVPPPPAQPQPSVQHHQHHPSSAMSAIRGTPVTDPLALSQHQLHAHQQQQH

QQQQQHVLTSHSMSSALGIKHEYGLTNL

>SACA_001219-T1 pb
MASDETGFIGSQPSMAEYMSSINPLLVDIDVPASVAAVEAMATSVSAAENTGEYAWMKEKKSQKKPP
PVVLQENGLPRRLRTAYTNTQLLELEKEFHFNKYLCRPRRIEIAAALDLSERQVKVWFQNRRMKHKRQ
SLSKTGGGSSGSVAGDRAVGDGGDGDVVSEDDNAGQDFKICAHSKPRNEEYDANDSLTKQERKLDL
DADKEVSDLKVSVSDMQCSSADEANSNGRCTRRIAVKIIGESGFIQKTAGFNQSTARAQLSSPLAECD
KKCQQSPSSIRASSSPYDRTSAVPMPASMSPGVWGQTAYHHKSFPESNSHILAEYNHGVRHMDNEA
ANSTDFNANIASQAADHHQVMNGQSAMNNEACVTLPRQQNIYQSQDSYSKQAGMNHQSLYSRESY
GEYHNYTPDETVSMPAIGVSADSSKTAGYHQHQTLGYHGYQQHAQDNIQQQHFHQQHQNQVMSTTI
AAYQTSYPETNYPGYDQYDQYCGYEQNYYFTEGAANDADTNVLTHGLANQATPAHCYAPIMQYNAF
DASVHERMQPNAMPEKHNQSADDFHLNHLTMAATEDTIDSGADHVKKSKAAAARSQAMEGTLAASI
NTYLGYGDQYSDSVETMPLTDGSGESVEFSCYDTTQSSPADFNFLSKLMSDDSSHCLGSTCKQC

>SACA_010140-T1 lab
MTNIPKVFAHHLEAPQVGLVCIADTIRWPSESAVQRGRMNGVQKQSVKELRIGVEGVEHTDTPSCTQ
PHGASTRTPTGSATLIDHDRVAIDPIDVVASSGHRSGDCITKRLPDPTTTKPPTDWYLLKKSAVMNSG
VYSSCGVDASAVAAGYQHAAAAYGGGAGEMLYYPAVAADAGAAAVSGPYLMTSAMAGVQAEPHSA
PMTNYTALDDYQQPSVHLHHADQQQQHSAVATAAYHSAMAQLQPNSAVTATTTLDASVGYRSHFKQ
HHQQHQQEYPAALPAVVLTGESAASVGATLPSYPASYVDGHAAMGMYGARSHMVAGCYMSQLRDH
AVPPRRAAPPPAAPTYKWMQVKRNNPRPVPKTTEFSFGAAGLNNTGRTNFTTKQLTELEKEFHFNKY
LTRARRIEIATALALNETQVKIWFQNRRMKQKKRIKEGLIPQQTLSTSPSSMSVSPLGVSRTDGSADKQ
MSPLPAKIEASPSPASVSPR

Sequences of *S. carcini* otopetrin

>SACA_015844-T1 otopetrin_1
MVGLESPVGMACVNADNTQTFSEARAKLLHSANRAERRQLVDNWWRNRKVSDKCGQLEDNSSSM
PRFPSRILKEIPYIDGVGDKEMADCSIDLVEVRHPTVSVLKMTPERPRSRFASQNKTAPIKPKRWPLCC
PVAVGERSGDSGVAGVGGGGAASCPMAMDTADVPLATVDSIDSEAGEYIDNLGAAAGSAAGADHAL
DHVVDTATLPAGVVDIPSPPPPVASGVLCNRIGTPMLEVGGAGGAAACSLKSVSGATGKTACFRIFSV
VYALAAVASCLVFVITEIITNKIPLYFFEVFFTYMYGASVLFLLYVFIYLLHESSTKVGRKRQGVGCCGKK
SKSTSADEHLNEAGSPEKLTKEWKPYKTKTSDSEHSHGGFFLRIGAIAFGLGTMVYNGMELGMFFEIP
SSSQCWQILMAVNPCLQATFTFMQMYFIFMNSRLNIHKFKCLARFGLMHMIATNVCVWIRTVVRESLR
VYTDYNSANNLKMTQDFLVLGGGGSEFSCNRTDIMGSIVNDASPYLFPLIVEYSLIGAAVLYVMWENIG
RCPRYLEEDDIPDHISVTSRKAHTKVDCIGASKGLFCGLLVCVAAIISLVLYFVLIDHKQKKHRQLAIFLA
DTSHASILLLMIIGALIGCCRNRKLKFVHDKPDILNAILLRVSAFGLFVYSAFCVLSAAVSPLPDVPNLLVL
VASGMAILQVLVQLLYVSDVSKRCVYLPEHDLTKPGRQMVTFMLLCNLALCAVYTFEIDKVRANPVQL
NFYGSLNWAVILRVTLPLAIFHRFYSAVVLAEVWKSSYKARAD

>SACA_012179-T1 otopetrin_2
MSHQQQAKTEEDPLRTQTRENMVRNRAASKAIQHFNKIAEDGAESDRHKVAGPHICRRHREAAKSR
AERHSSSLIGATSLNGGGAVEVHAERRKSAHQRRTASADEEFARRTSFRQQVLSRPRAETGSVANG
VATKTATSKTVGPREATESSNDVLSESYVVVPDELVTAKSPQVASPPPNVPVKKSLAKKTKEEATRRG
PTVEQLWPKSWGVYSDNPDRDSLDGHLPSSGHGTNTTHHPRACVDTDAASNVGADAGSGGRRATP
GAAVNNKKKSSPSTPLPTSVTVAKRAGTPDALADRESALRMCSPVDPESGNNSTMAGQVSRTNDFS
HVALSQMWRSLSCMYGKLVVLVSVAFCVTEVLDTSIIPFTYQGVFLMCLYVVGIVTILCIYISVVADSLS
SVSSSQQNLTASCSAGGAVSAADLTSLASFGTLKRAHISRRKVNRSSFYLRIGALLFGTGTLIFNGLEIA
MHSTSNTNCSGDMAFAHPILQAIFTFLQMHFLFVNSEVVVEKLGNLARFGLVHLVATNLSVWFRMVIW
DSANDWIHFAHAYANAKTSNPTELPPRAIRLQGFPGLKDLGYNGYIDLSSADSTTEVNRLGPVDAGVL
YYGNPFSAAAVTYIMWTSVGDTRMKKAVTKKLRASMGTLDMKTPAQRNTDCQSASKGLFFGLLCLVS
GIVVLIIFFVMKDTSTFEEHMFWLSSGTQLVVLTLSVSMTIVGFHQIPKLSLQHNRQPTPLDKMLSSVTM
FGVYLIGVFGMIVGGLNITESRHMALFAINTVLIIQATLQGMLVSEAAMRHCATKQQQTFKPGRQIITFL
MFSNVTLWMLDTLMAHHWLAHEMQSSYYGFLTWGVISRIGLPLMIFYRFHQAVVLVGIWKKSYKTRID

>SACA_006178-T1 SACA_006178
MADCEMCTVTGWQIGFWVSSRWTRRCFSWGPQPLQAPPWRCHCPENTPLLSISLHVVFCLIEQGV
MMLGVMMLGVMMLVCCLIEQGVMMLVFCLIEQGVMMLVCCLIEQGVMMLWTTCGRSSIMGNLVNAV
SPFLFPCSIEYSLICAATLYIMWKHLNKLNTVYLERHPSLDNARKHHYSVDCASASKGLFLGILVFVATVI
SLILFFVLIDKESTKQAAIKSAGITELVLYTLTSTAIIIGTIQVQKLSYHANRNIELDTILLVAAQTGIYLYACF
DIIGAYFSFNIMSLITGIMSVVQVTLQTMFILDTTHRQAFSSKQVLHDFSHSTCLRVRVGPPNRGFEKTE
K

Sequences of S.carcini Neurohormones & juvenile hormone binding protein (JHBP)

>SACA_003990-T1 crust_neurohorm_1
MTPDMVCPPQAFTFSSLTRPLSQSSQTPRRTQTITVLLYLATGFVLLAAIVPRAECMHIERGDFVGRAL
TRQAFDDLSCNGTFNSTLFSTLNSICNRCYWLYQEPEYHGLCRSECFTTSYFHGCLVVRMMEESAAK
YSADIAKFAGRPLTFLHPDNINHTRRSFNISSNL

>SACA_005141-T1 crust_neurohorm_2
MARVTSVLVLSALTATWLAAFGSAASPYFTRLSVAQHTANHGPVKRHFHQIGCLGVHDESRMTKLELI
CDQCYELYQEPELHGLKDCFASDYFIGCIEALLMTSQQEELMGHVRYLKGGDK

>SACA_006180-T1 JHBP
MAIFRTGVCVLCLLLVLCVLPVIHSRGHSSYNDIQHSGSGIDPQLLRNLVSPHEIARLIEEEILARRAMLP
ELICGGLPSLGIPPLDPVSVGDVPVNFTMDVVTLEGNFSEVQLEGLSEVRLTNVHLQLPGMLLQLDMR
LPSLVVKGKYLLGGSFSSLISLSGSGPLTLTLTDVDVRLICQVRLYYDRVHIARVDFTIAVANSSVQLVG
YLKEVGFEDLAQQFLEDMSGDFLRTAIMRFREDQFPNMVQRANRFLRSFDWRQRLPDAIALFLGFGA
NSGGGYNAYTPTVTNNNLVDRFLMSLRSHFKNNAFDLPNLDILTKKKFFKWTLNNGVRLLPGKMFGL
DSLSSVGDVLISRHNHIFTFNLDLAAYNLKVIFPSQLIVINKNKKFITFAHISRIKINVQLQLDSQYLSLQLT
ALHIIHIGDIRVELSGLGALEVVAAPVAEYKVNLEQSKRQMARQAAKIGGGLLASALANVDINSLLTDLV
NGHVYAVDPGIVFPDQQTNMTARFNNLLSSAMGVNDSGGQAADFGSLLDLFLGGSDDDDQDTDQTL
DDLDPDILAEFGLSVDSLSDTNSASNLETNPAINQSMKSTSSTNESLLGNTISATGDVSAVSTESSSLS
AASAPGNGRQSDAAASPRATAASSTTPDPLDAIDPDILAAFGLT

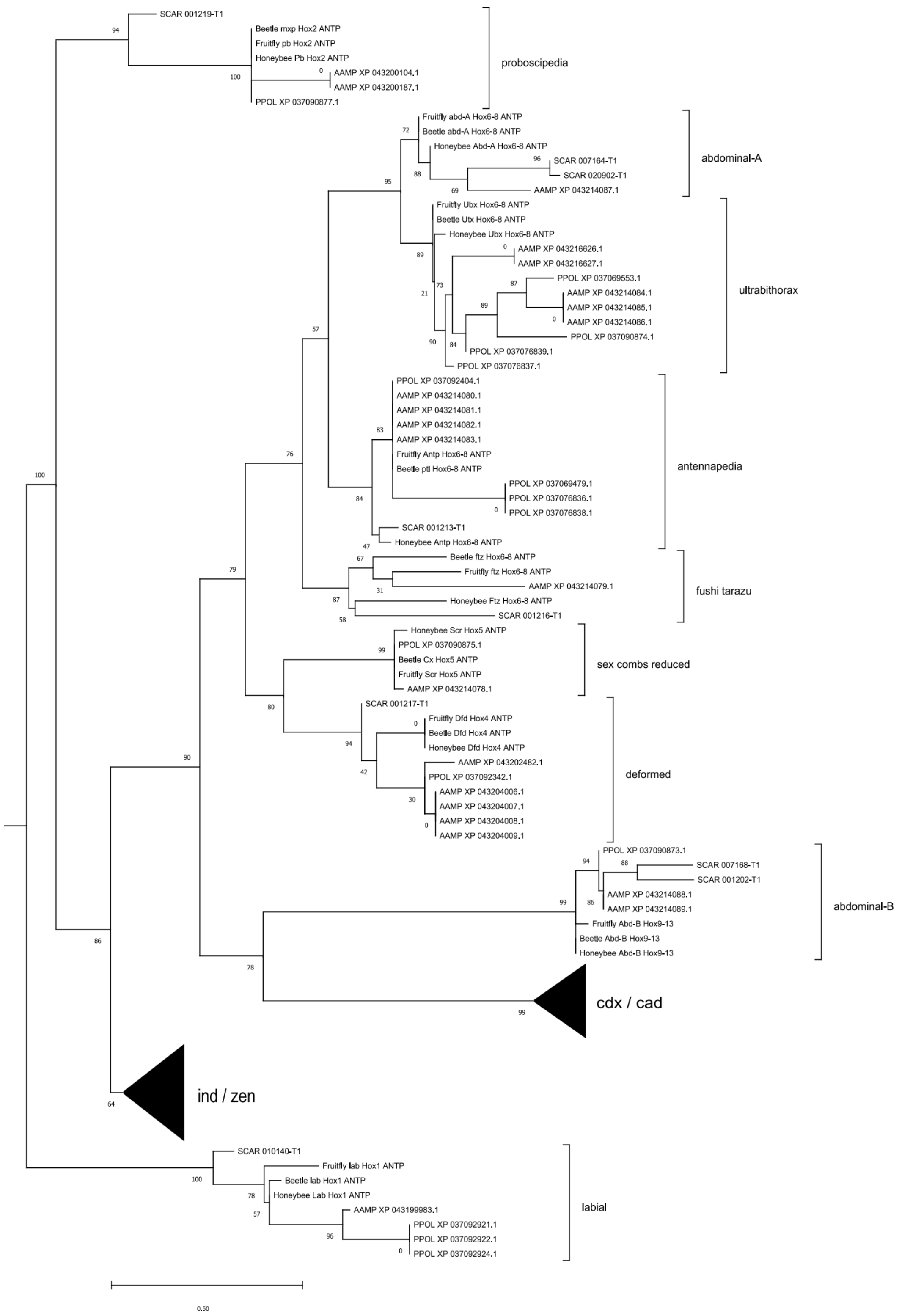

Supplementary figure 1. Tree of hox gene domains (subtree from full homeobox containing protein sets). PPOL = P. pollicipes, AAMP = A. amphitrite, SCAR= S. carcini. Fruitfly, beetle

and honeybee sequences were downloaded from the homeobox database (http://homeodb.zoo.ox.ac.uk). Numbers are bootstrap values from FastTree analysis.

Supplementary table 1. Assembly statistics of the Sacculina carcini genome (this study) and other available Cirrpedia genomes, contig numbers according to quast (broken on multiple Ns)

Assembly	*A. amphitrite*		*S. balanoides*		*P. pollicipes*		*S. carcini*	
	**scaff.**	**cont.**	**scaff.**	**cont.**	**scaff.**	**cont.**	**scaff.**	**cont.**
No. of contigs	2240	3312	16596	18316	1254	NA	13055	13532
No. of contigs >1 kbp	2240	3312	16477	18316	1187	11970	11217	11686
No. of contigs >10 kbp	2184	3172	10691	12169	923	10810	3339	3733
Largest contig (Mbp)	2.224	1.452	1.400	0.603	64.044	0.802	1.562	1.562
Total length (Mbp)	613.41	610.57	486.01	482.35	770.09	761.77	298.35	297.15
Total length (Mbp) (contigs >10 kbp)	613.09	609.78	451.80	445.89	769.00	755.11	268.79	267.32
N50	458238	313401	56748	45551	47 Mb	109725	161259	123767
N75	254816	177769	24497	21768	37 Mb	55851	48672	42943
L50	415	605	1895	2728	8	2074	455	590
L75	861	1248	5178	6576	12	4465	1295	1608
No. of N's per 100kbp	464.29	0.17	735.70	1.89	1079	0	402.65	0.30

Supplementary table 2. Accession numbers and genome size of genome assemblies used in the comparative analyses.

**Species**	**Higher ranking taxa**	**NCBI genome accession**	**Genome size (Mbp)**
Tribolium castaneum	Hexapoda - Coleoptera	GCA_000002335.3	166
Apis mellifera	Hexapoda - Hymenoptera	GCF_003254395.2	225
Schistocerca americana	Hexapoda - Orthoptera	GCF_021461395.2	8990
Ischnura elegans	Hexapoda - Odonata	GCF_921293095.1	1723
Darwinula stevensoni	Crustacea - Ostracoda	GCA_905338385.1	382
Notodromas monacha	Crustacea - Ostracoda	GCA_905338405.1	377
Portunus trituberculatus	Crustacea - Decapoda	GCF_017591435.1	1005
Homarus americanus	Crustacea - Decapoda	GCF_018991925.1	2292
Procambarus clarkii	Crustacea - Decapoda	GCF_020424385.1	2735
Penaeus japonicus	Crustacea - Decapoda	GCF_017312705.1	1705
Trinorchestia longiramus	Crustacea - Amphipoda	GCA_006783055.1	886
Daphnia pulex	Crustacea - Phyllopoda	GCF_021134715.1	133
Lepeophtheirus salmonis	Crustacea - Copepoda	GCA_001005205.1	665
Eurytemora affinis	Crustacea - Copepoda	GCA_000591075.2	389
Tigriopus californicus	Crustacea - Copepoda	GCA_007210705.1	191
Amphibalanus amphitrite	Crustacea - Cirripedia	GCA_009805615.1	613
Pollicipes pollicipes	Crustacea - Cirripedia	GCA_011947565.2	770

Supplementary table 3. Orthogroups that exhibit notable expansion in S. carcini (SACA) in comparison to a barnacle (AAMP, Amphibalanus amphitryte) and four other crustacean species (EAFF,HAZT,LSAL,PVAN)

Orthogroup	EAFF	HAZT	LSAL	PVAN	AAMP	SACA	PFAM ID	domain name
OG0000214	2	6	1	3	2	48	PF00069.25	Pkinase
OG0000013	18	7	13	13	13	30	PF07714.17	Pkinase_Tyr
OG0002381	1	0	0	0	0	27	PF00105.18	zf-C4
OG0000370	8	3	2	6	7	12	PF01576.19	Myosin_tail_1
OG0002590	1	1	1	1	2	11	PF02759.19	RUN
OG0000540	4	2	4	4	3	11	PF02210.24	Laminin_G_2
OG0001168	1	1	0	1	1	10	PF00621.20	RhoGEF
OG0000565	6	6	1	1	3	10	PF06459.12	RR_TM4-6
OG0000511	1	2	2	2	6	10	PF00060.26	Lig_chan
OG0001100	4	1	0	1	4	9	PF00042.22	Globin
OG0000938	3	2	1	1	2	9	PF08326.12	ACC_central
OG0000820	3	2	2	3	4	9	PF03712.15	Cu2_monoox_C
OG0000364	12	9	3	5	2	9	PF00501.28	AMP-binding
OG0000151	8	3	7	5	2	9	PF00104.30	Hormone_recep
OG0002426	1	1	1	1	2	8	PF04811.15	Sec23_trunk
OG0001010	3	1	1	1	4	8	PF07714.17	Pkinase_Tyr
OG0000739	6	1	2	3	3	8	PF16482.5	Staufen_C
OG0000581	1	3	2	3	3	8	PF00130.22	C1_1
OG0005021	1	0	1	0	1	7	PF04931.13	DNA_pol_phi
OG0003136	1	1	1	2	3	7	PF12816.7	Vps8
OG0002024	1	1	1	2	1	7	PF01762.21	Galactosyl_T
OG0000520	8	1	1	1	2	7	PF02124.15	Marek_A
OG0000315	7	5	3	6	2	7	PF02124.15	Marek_A
OG0005527	0	2	0	2	1	6	PF12736.7	CABIT
OG0004259	1	1	1	3	2	6	PF16529.5	Ge1_WD40
OG0002948	1	1	1	1	1	6	PF16842.5	RRM_occluded
OG0002678	3	1	1	4	1	6	PF08506.10	Cse1
OG0002656	1	2	1	2	1	6	PF07714.17	Pkinase_Tyr
OG0002577	0	0	0	0	0	6	PF07020.11	Orthopox_C10L
OG0002447	0	4	1	4	1	6	PF17874.1	TPR_MalT
OG0002153	1	2	1	1	3	6	PF02145.15	Rap_GAP
OG0002084	2	2	0	1	2	6	PF03131.17	bZIP_Maf
OG0002022	9	1	1	4	1	6	PF00615.19	RGS
OG0001990	4	2	1	3	1	6	PF02825.20	WWE
OG0001891	0	2	2	2	2	6	PF05889.13	SepSecS
OG0001846	1	1	1	3	2	6	PF09330.11	Lact-deh-memb
OG0001693	1	2	1	2	0	6	PF08514.11	STAG
OG0001685	4	1	3	3	2	6	PF15304.6	AKAP2_C
OG0001153	2	2	1	2	2	6	PF03062.19	MBOAT
OG0001152	2	1	1	2	2	6	PF00083.24	Sugar_tr
OG0001125	1	2	1	1	2	6	PF08694.11	UFC1
OG0000601	2	2	1	2	2	6	PF00630.19	Filamin
OG0000499	5	3	3	3	2	6	PF00060.26	Lig_chan
OG0000316	9	2	4	1	1	6	PF00084.20	Sushi
OG0009221	0	0	0	1	0	5	PF14580.6	LRR_9
OG0008430	0	0	0	0	0	5	PF06524.12	NOA36
OG0002398	1	1	2	5	1	5	PF15015.6	NYD-SP12_N

OG0004988	1	2	1	1	2	5	PF03133.15	TTL
OG0004400	1	2	1	1	1	5	PF17003.5	Actin_micro
OG0004295	0	1	1	1	0	5	PF02347.16	GDC-P
OG0004175	1	1	1	2	1	5	PF14872.6	GHL5
OG0004142	1	2	1	1	1	5	PF12689.7	Acid_PPase
OG0004119	1	1	1	1	1	5	PF00454.27	PI3_PI4_kinase
OG0003866	1	1	1	2	1	5	PF00780.22	CNH
OG0003859	2	1	2	1	2	5	PF16881.5	LIAS_N
OG0003349	3	1	1	2	1	5	PF00158.26	Sigma54_activat
OG0002628	1	1	1	1	1	5	PF05029.13	TIMELESS_C
OG0002506	1	1	1	1	2	5	PF11819.8	DUF3338
OG0002463	1	3	1	1	1	5	PF00248.21	Aldo_ket_red
OG0002460	1	2	1	1	1	5	PF00557.24	Peptidase_M24
OG0002053	1	1	1	2	0	5	PF00055.17	Laminin_N
OG0002032	2	2	2	3	1	5	PF00176.23	SNF2_N
OG0002023	2	2	1	2	1	5	PF00640.23	PID
OG0001916	2	2	1	1	1	5	PF09790.9	Hyccin
OG0001793	5	2	1	1	1	5	PF14228.6	MOR2-PAG1_mid
OG0001665	2	1	1	6	1	5	PF06990.11	Gal-3-0_sulfotr
OG0001637	2	1	1	1	1	5	PF03166.14	MH2
OG0001583	2	3	1	3	1	5	PF08696.11	Dna2
OG0001341	4	1	3	1	1	5	PF03351.17	DOMON
OG0001247	1	3	1	2	1	5	PF14719.6	PID_2
OG0001107	3	1	1	1	1	5	PF00791.20	ZU5
OG0001037	3	3	1	5	1	5	PF10567.9	Nab6_mRNP_bdg
OG0000999	2	2	1	6	1	5	PF17743.1	DUF5580
OG0000951	7	6	1	2	1	5	PF14776.6	UNC-79
OG0013273	0	0	0	0	0	4	PF04064.13	DUF384
OG0007720	1	1	1	1	0	4	PF16975.5	UPAR_LY6_2
OG0007658	0	0	2	3	1	4	PF02932.16	Neur_chan_memb
OG0006668	1	1	1	1	0	4	PF05997.12	Nop52
OG0006539	1	1	1	0	1	4	PF03914.17	CBF
OG0006531	1	0	1	1	1	4	PF16652.5	PH_13
OG0006023	1	2	1	1	1	4	PF03372.23	Exo_endo_phos
OG0005329	1	1	1	1	1	4	PF06984.13	MRP-L47
OG0005151	1	1	1	2	1	4	PF00687.21	Ribosomal_L1
OG0005106	1	2	0	1	1	4	PF04762.12	IKI3
OG0005100	1	1	1	1	1	4	PF00370.21	FGGY_N
OG0005082	1	1	1	1	1	4	PF02114.16	Phosducin
OG0004834	1	1	1	0	1	4	PF00632.25	HECT
OG0004821	1	0	1	2	1	4	PF17825.1	DUF5587
OG0004741	2	1	0	1	0	4	PF00387.19	PI-PLC-Y
OG0004661	0	1	0	1	1	4	PF01553.21	Acyltransferase
OG0004639	1	1	1	1	1	4	PF07393.11	Sec10
OG0004556	1	2	1	1	1	4	PF00595.24	PDZ
OG0004545	1	1	1	1	1	4	PF14597.6	Lactamase_B_5
OG0004502	1	1	1	1	1	4	PF12584.8	TRAPPC10
OG0004363	1	1	1	1	1	4	PF00454.27	PI3_PI4_kinase

OG0004351	1	1	1	2	1	4	PF07502.14	MANEC
OG0004303	1	3	1	1	0	4	PF13774.6	Longin
OG0004251	1	1	1	3	1	4	PF01585.23	G-patch
OG0004152	1	1	1	0	1	4	PF18386.1	ROQ_II
OG0004114	1	2	1	1	1	4	PF17136.4	ribosomal_L24
OG0004014	1	0	1	1	1	4	PF07970.12	COPIIcoated_ERV
OG0003764	1	2	1	1	1	4	PF00380.19	Ribosomal_S9
OG0003744	1	1	1	2	1	4	PF00928.21	Adap_comp_sub
OG0002907	2	1	1	1	1	4	PF05783.11	DLIC
OG0002900	3	1	0	3	1	4	PF07714.17	Pkinase_Tyr
OG0002358	1	1	1	1	1	4	PF00069.25	Pkinase
OG0001927	1	1	2	1	1	4	PF00568.23	WH1
OG0010579	0	0	0	0	1	3	PF01061.24	ABC2_membrane
OG0009409	1	0	0	1	1	3	PF06247.11	Plasmod_Pvs28
OG0007867	1	1	0	1	1	3	PF06128.11	Shigella_OspC
OG0007027	0	1	0	2	1	3	PF01808.18	AICARFT_IMPCHas
OG0006826	1	0	0	3	1	3	PF06384.11	ICAT
OG0006700	2	1	1	1	0	3	PF07956.11	DUF1690
OG0006674	1	1	1	1	0	3	PF13328.6	HD_4
OG0006538	2	2	1	1	1	3	PF00644.20	PARP
OG0006518	1	1	1	1	1	3	PF00046.29	Homeodomain
OG0006282	1	1	1	1	1	3	PF01267.17	F-actin_cap_A
OG0006150	2	1	0	1	1	3	PF02792.14	Mago_nashi
OG0006140	1	1	1	1	1	3	PF17034.5	zinc_ribbon_16
OG0006112	0	1	1	2	1	3	PF06047.11	Nkap_C
OG0006102	1	1	1	1	1	3	PF01399.27	PCI
OG0006098	1	1	1	1	1	3	PF00648.21	Peptidase_C2
OG0006069	1	2	1	1	1	3	PF00198.23	2-oxoacid_dh
OG0006048	1	1	1	1	1	3	PF00227.26	Proteasome
OG0005944	1	1	0	1	1	3	PF12756.7	zf-C2H2_2
OG0005892	1	0	1	3	1	3	PF07890.12	Rrp15p
OG0005835	0	1	0	1	1	3	PF06888.12	Put_Phosphatase
OG0005832	1	1	0	1	1	3	PF01712.19	dNK
OG0005800	2	1	1	1	1	3	PF09398.10	FOP_dimer
OG0005708	1	1	0	2	0	3	PF03635.17	Vps35
OG0005690	0	2	1	2	0	3	PF01216.17	Calsequestrin
OG0005659	1	1	1	1	1	3	PF01434.18	Peptidase_M41
OG0005563	1	2	1	1	1	3	PF00152.20	tRNA-synt_2
OG0005555	1	2	0	1	1	3	PF00472.20	RF-1
OG0005504	1	1	1	1	1	3	PF17121.5	zf-C3HC4_5
OG0005308	1	1	0	1	1	3	PF06003.12	SMN
OG0005185	1	1	1	1	0	3	PF03615.15	GCM
OG0005169	0	0	0	0	1	3	PF07782.13	DC_STAMP
OG0005156	1	1	1	2	1	3	PF16399.5	Aquarius_N
OG0005149	1	1	1	3	1	3	PF10156.9	Med17
OG0005126	1	1	1	1	1	3	PF08083.11	PROCN
OG0005121	4	1	0	1	1	3	PF06644.11	ATP11
OG0005059	0	1	0	1	1	3	PF01040.18	UbiA

OG0005041	2	1	1	2	1	3	PF04707.14	PRELI
OG0005009	1	1	3	0	1	3	PF04157.16	EAP30
OG0005008	1	1	1	2	1	3	PF12430.8	ABA_GPCR
OG0005000	2	1	1	2	1	3	PF01189.17	Methyltr_RsmB-F
OG0004997	1	1	2	2	1	3	PF10152.9	CCDC53
OG0004932	3	2	1	2	1	3	PF07969.11	Amidohydro_3
OG0004877	1	1	1	1	1	3	PF02124.15	Marek_A
OG0004823	1	2	1	2	1	3	PF09430.10	DUF2012
OG0004686	2	1	1	2	1	3	PF01248.26	Ribosomal_L7Ae
OG0004658	1	1	1	3	1	3	PF08519.12	RFC1
OG0004654	1	1	0	1	1	3	PF07714.17	Pkinase_Tyr
OG0004651	1	0	1	2	1	3	PF08613.11	Cyclin
OG0004540	3	1	0	2	1	3	PF15023.6	DUF4523
OG0004496	1	1	1	2	1	3	PF01248.26	Ribosomal_L7Ae
OG0004442	1	1	2	1	1	3	PF01694.22	Rhomboid
OG0004394	2	1	0	1	1	3	PF09749.9	HVSL
OG0004381	2	2	1	1	1	3	PF00206.20	Lyase_1
OG0004353	1	1	1	1	1	3	PF02799.15	NMT_C
OG0004288	2	1	1	1	0	3	PF15510.6	CENP-W
OG0004279	1	1	1	2	1	3	PF16529.5	Ge1_WD40
OG0004262	1	3	2	1	1	3	PF16319.5	DUF4958
OG0004248	1	1	1	2	1	3	PF03796.15	DnaB_C
OG0004232	0	1	0	1	1	3	PF03062.19	MBOAT
OG0004212	1	1	1	2	1	3	PF07842.12	GCFC
OG0004189	1	1	1	2	1	3	PF00071.22	Ras
OG0004187	1	1	1	1	1	3	PF05679.16	CHGN
OG0004169	0	2	1	1	1	3	PF03643.15	Vps26
OG0004151	1	1	1	1	1	3	PF04762.12	IKI3
OG0004141	3	3	1	1	1	3	PF00899.21	ThiF
OG0004118	1	2	1	1	1	3	PF02785.19	Biotin_carb_C
OG0004053	1	1	1	2	1	3	PF01207.17	Dus
OG0003966	3	1	0	2	1	3	PF01746.21	tRNA_m1G_MT
OG0003879	2	1	1	2	1	3	PF07933.14	DUF1681
OG0003868	1	1	1	1	1	3	PF14580.6	LRR_9
OG0003858	4	1	1	1	1	3	PF01363.21	FYVE
OG0003836	1	1	1	1	1	3	PF12074.8	Gcn1_N
OG0003835	1	0	1	3	1	3	PF11027.8	DUF2615
OG0003828	3	1	1	2	1	3	PF00179.26	UQ_con
OG0003821	0	1	1	4	1	3	PF02229.16	PC4
OG0003811	1	1	1	2	1	3	PF03947.18	Ribosomal_L2_C
OG0003781	0	2	1	1	1	3	PF12906.7	RINGv
OG0003757	1	1	1	7	1	3	PF05891.12	Methyltransf_PK
OG0003749	1	1	1	1	1	3	PF00171.22	Aldedh
OG0003662	1	1	1	1	1	3	PF02862.17	DDHD
OG0003655	1	2	1	1	1	3	PF16529.5	Ge1_WD40
OG0003581	1	2	1	5	0	3	PF03462.18	PCRF
OG0003573	2	2	1	2	0	3	PF07377.12	DUF1493
OG0003560	1	1	1	2	0	3	PF02615.14	Ldh_2

(Supplementary table 3, continued)

OG0003546	3	1	1	3	1	3	PF03747.14	ADP_ribosyl_GH
OG0003522	2	1	0	2	1	3	PF00683.17	TB
OG0003488	1	2	1	1	1	3	PF18136.1	DNApol_Exo
OG0003431	1	1	1	2	1	3	PF06128.11	Shigella_OspC
OG0003345	2	1	1	2	1	3	PF13561.6	adh_short_C2
OG0003322	1	2	1	1	1	3	PF00069.25	Pkinase
OG0003318	1	1	0	1	1	3	PF04045.14	P34-Arc
OG0003179	1	1	1	5	1	3	PF16206.5	Mon2_C
OG0003150	3	1	1	2	1	3	PF03635.17	Vps35
OG0003114	1	3	1	1	1	3	PF01168.20	Ala_racemase_N
OG0003092	2	1	1	2	1	3	PF01813.17	ATP-synt_D
OG0003079	1	1	1	1	1	3	PF00069.25	Pkinase
OG0003077	2	0	1	2	1	3	PF00622.28	SPRY
OG0002989	1	1	1	2	0	3	PF01803.16	LIM_bind
OG0002979	1	3	1	6	0	3	PF13523.6	Acetyltransf_8
OG0002942	2	1	1	1	1	3	PF02450.15	LCAT
OG0002937	2	1	1	1	1	3	PF01467.26	CTP_transf_like
OG0002930	2	2	2	1	1	3	PF14954.6	LIX1
OG0002896	1	1	1	1	1	3	PF15099.6	PIRT
OG0002890	3	1	2	3	1	3	PF00122.20	E1-E2_ATPase
OG0002883	3	1	1	3	1	3	PF02800.20	Gp_dh_C
OG0002767	1	1	1	1	1	3	PF05002.15	SGS
OG0002727	2	1	2	1	1	3	PF00297.22	Ribosomal_L3
OG0002591	1	1	1	2	1	3	PF16669.5	TTC5_OB
OG0002376	1	1	1	2	0	3	PF04678.13	MCU
OG0002210	1	1	1	0	1	3	PF01733.18	Nucleoside_tran
OG0002168	1	1	1	2	1	3	PF00410.19	Ribosomal_S8
OG0001848	3	1	1	1	1	3	PF16389.5	DUF4998
OG0001795	1	1	1	3	1	3	PF04987.14	PigN
OG0001774	1	1	1	12	1	3	PF05154.16	TM2
OG0001725	1	1	1	4	1	3	PF13901.6	zf-RING_9
OG0001509	2	1	1	2	0	3	PF05622.12	HOOK
OG0000878	3	1	1	1	1	3	PF00850.19	Hist_deacetyl
OG0000855	1	1	0	2	1	3	PF00013.29	KH_1

Supplementary table 4. Orthogroups that exhibit notable contraction or total reduction in S. carcini (SACA) in comparison to a barnacle (AAMP, Amphibalanus amphitryte) and four other crustacean species (EAFF,HAZT,LSAL,PVAN)

Orthogroup	EAFF	HAZT	LSAL	PVAN	AAMP	SACA	PFAM ID	domain name
OG0000127	13	6	4	7	40	7	PF13423.6	UCH_1
OG0000016	3	35	6	38	37	14	PF00685.27	Sulfotransfer_1
OG0000008	21	29	9	34	28	11	PF00067.22	p450
OG0000020	21	13	11	26	21	9	PF00083.24	Sugar_tr
OG0000004	25	38	25	63	44	9	PF00089.26	Trypsin
OG0001003	4	1	1	15	1	7	PF17005.5	WD40_like
OG0000067	17	13	13	11	22	6	PF00188.26	CAP
OG0000039	13	15	10	15	18	6	PF01607.24	CBM_14
OG0000134	3	2	2	18	38	5	PF06524.12	NOA36
OG0000049	7	17	4	39	11	4	PF11838.8	ERAP1_C
OG0000048	6	8	2	23	10	4	PF01400.24	Astacin
OG0000037	29	20	11	31	18	4	PF00063.21	Myosin_head
OG0000030	14	10	6	11	13	4	PF03089.14	RAG2
OG0000169	3	7	1	35	14	3	PF17172.4	GST_N_4
OG0000145	21	30	5	7	11	3	PF00069.25	Pkinase
OG0000095	7	5	4	14	11	3	PF00704.28	Glyco_hydro_18
OG0000011	14	14	7	28	17	3	PF00067.22	p450
OG0000198	3	2	1	0	73	2	PF04506.13	Rft-1
OG0000172	5	18	0	10	12	2	PF02087.15	Nitrophorin
OG0000120	6	8	2	12	13	2	PF00474.17	SSF
OG0000104	15	11	2	3	11	2	PF01306.19	LacY_symp
OG0000022	17	4	7	19	10	2	PF10129.9	OpgC_C
OG0000018	16	6	7	47	31	2	PF00135.28	COesterase
OG0000660	2	1	1	4	24	1	PF05186.13	Dpy-30
OG0000360	14	4	2	2	11	1	PF03803.15	Scramblase
OG0000357	1	1	2	1	37	1	PF00093.18	VWC
OG0000219	5	9	3	4	6	1	PF00135.28	COesterase
OG0000218	12	4	4	7	8	1	PF02180.17	BH4
OG0000147	8	16	2	7	8	1	PF16865.5	GST_C_5
OG0000113	9	15	5	14	9	1	PF02738.18	Ald_Xan_dh_C2
OG0000017	6	13	3	30	46	1	PF00147.18	Fibrinogen_C
OG0007216	0	0	0	0	9	0	PF00078.27	RVT_1
OG0006767	0	0	0	0	12	0	PF00336.18	DNA_pol_viral_C
OG0004049	2	0	0	2	10	0	PF03321.13	GH3
OG0003304	1	2	0	3	5	0	PF00154.21	RecA
OG0003028	2	1	0	2	7	0	PF00083.24	Sugar_tr
OG0002946	3	3	1	2	4	0	PF03109.16	ABC1
OG0002921	1	2	1	5	2	0	PF14792.6	DNA_pol_B_palm
OG0002863	1	2	0	5	4	0	PF05986.14	ADAM_spacer1
OG0002692	3	5	0	2	2	0	PF00245.20	Alk_phosphatase
OG0002541	0	3	1	2	3	0	PF00445.18	Ribonuclease_T2
OG0002521	1	4	0	3	2	0	PF03189.13	Otopetrin
OG0002504	2	2	1	3	5	0	PF03089.14	RAG2
OG0002425	0	3	0	1	8	0	PF00305.19	Lipoxygenase
OG0002414	1	2	1	3	3	0	PF02129.18	Peptidase_S15
OG0002393	0	0	0	0	27	0	PF00147.18	Fibrinogen_C
OG0002262	1	1	2	1	3	0	PF01576.19	Myosin_tail_1
OG0002246	0	3	1	0	18	0	PF03184.19	DDE_1
OG0002108	1	2	1	2	4	0	PF05428.11	CRF-BP

(Supplementary table 4, continued)

OG0002101	1	2	4	2	3	0	PF06079.11	Apyrase
OG0002011	2	3	0	5	3	0	PF11017.8	DUF2855
OG0001723	3	2	2	3	5	0	PF00086.18	Thyroglobulin_1
OG0001546	2	9	0	1	8	0	PF13561.6	adh_short_C2
OG0001411	5	4	2	2	2	0	PF02453.17	Reticulon
OG0001331	2	4	3	10	2	0	PF12897.7	Aminotran_MocR
OG0001300	2	6	2	6	2	0	PF09235.10	SAM_Ste50p
OG0001268	4	1	0	4	2	0	PF06441.12	EHN
OG0001259	0	3	1	2	2	0	PF00795.22	CN_hydrolase
OG0001155	10	1	1	6	3	0	PF14582.6	Metallophos_3
OG0001127	3	2	2	5	2	0	PF01369.20	Sec7
OG0001092	2	1	1	2	5	0	PF05478.11	Prominin
OG0001091	3	3	2	6	7	0	PF07810.13	TMC
OG0001029	1	3	0	9	11	0	PF04199.13	Cyclase
OG0000900	4	3	2	4	2	0	PF00854.21	PTR2
OG0000897	3	3	4	3	4	0	PF13853.6	7tm_4
OG0000749	2	2	4	11	2	0	PF08005.12	PHR
OG0000713	6	1	2	5	2	0	PF12330.8	Haspin_kinase
OG0000709	7	0	3	7	3	0	PF17738.1	DUF5575
OG0000701	2	2	1	4	3	0	PF01697.27	Glyco_transf_92
OG0000694	2	2	0	5	4	0	PF01266.24	DAO
OG0000622	5	2	1	1	4	0	PF06027.12	SLC35F
OG0000593	5	8	0	3	4	0	PF03935.15	SKN1
OG0000490	1	6	0	3	4	0	PF13417.6	GST_N_3
OG0000417	4	4	3	8	6	0	PF01421.19	Reprolysin
OG0000358	0	5	1	9	9	0	PF04827.14	Plant_tran
OG0000343	2	5	2	2	5	0	PF00501.28	AMP-binding
OG0000330	0	0	0	1	9	0	PF07592.11	DDE_Tnp_ISAZ013
OG0000298	1	1	1	1	61	0	PF12348.8	CLASP_N
OG0000161	9	5	4	22	3	0	PF13019.6	Sde2_N_Ubi
OG0000046	6	13	1	30	7	0	PF04827.14	Plant_tran
